# Biobased Elastomer Nanofibers Guide Light‐Controlled Human‐iPSC‐Derived Skeletal Myofibers

**DOI:** 10.1002/adma.202110441

**Published:** 2022-03-31

**Authors:** Aimee Cheesbrough, Fabiola Sciscione, Federica Riccio, Peter Harley, Lea R'Bibo, Georgios Ziakas, Arnold Darbyshire, Ivo Lieberam, Wenhui Song

**Affiliations:** ^1^ UCL Centre for Biomaterials in Surgical Reconstruction and Regeneration Department of Surgical Biotechnology Division of Surgery and Interventional Science University College London London NW3 2PF UK; ^2^ Centre for Gene Therapy and Regenerative Medicine MRC Centre for Neurodevelopmental Disorders Centre for Developmental Neurobiology Kings College London London SE1 9RT UK

**Keywords:** biobased elastomer nanofibers, electrospinning, human induced pluripotent stem cells, myogenic differentiation, optogenetics

## Abstract

Generating skeletal muscle tissue that mimics the cellular alignment, maturation, and function of native skeletal muscle is an ongoing challenge in disease modeling and regenerative therapies. Skeletal muscle cultures require extracellular guidance and mechanical support to stabilize contractile myofibers. Existing microfabrication‐based solutions are limited by complex fabrication steps, low throughput, and challenges in measuring dynamic contractile function. Here, the synthesis and characterization of a new biobased nanohybrid elastomer, which is electrospun into aligned nanofiber sheets to mimic the skeletal muscle extracellular matrix, is presented. The polymer exhibits remarkable hyperelasticity well‐matched to that of native skeletal muscle (≈11–50 kPa), with ultimate strain ≈1000%, and elastic modulus ≈25 kPa. Uniaxially aligned nanofibers guide myoblast alignment, enhance sarcomere formation, and promote a ≈32% increase in myotube fusion and ≈50% increase in myofiber maturation. The elastomer nanofibers stabilize optogenetically controlled human induced pluripotent stem cell derived skeletal myofibers. When activated by blue light, the myofiber–nanofiber hybrid constructs maintain a significantly higher (>200%) contraction velocity and specific force (>280%) compared to conventional culture methods. The engineered myofibers exhibit a power density of ≈35 W m^−3^. This system is a promising new skeletal muscle tissue model for applications in muscular disease modeling, drug discovery, and muscle regeneration.

## Graphical abstract

Elastomeric nanofiber sheets, made from a new biobased nanohybrid polymer, support the differentiation and contractile function of human induced pluripotent stem cell derived skeletal muscle fibers. The controlled alignment of nanofibers provides extracellular‐matrix‐like guidance, promoting myofiber differentiation and maturation. The hyperelastic properties of the elastomer nanofibers stabilize optogenetically activate contractions and promote culture longevity.

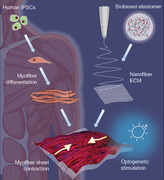

## 1 Introduction

Advances in the field of skeletal muscle tissue engineering depend on generating stable and life‐like skeletal muscle microtissues in vitro. This requires an interdisciplinary approach, whereby cells are incorporated into a biological or synthetic mechanical microenvironment. Such work enables accurate modeling of skeletal muscle function and disease, and progress in the generation of transplantable tissues to treat muscle trauma and degeneration. Skeletal muscle is a highly organized complex organ composed of connective tissue, blood vessels, and aligned contractile myofiber bundles, which are innervated by motor neurons (MNs); the output layer of the central nervous system. This complex network of different cell types and extracellular structures work in synergy to contribute to muscle force generation, transmission, maintenance, and repair.^[^ ^1^ ^]^

Scalable nanostructured biomaterial substrates are required to mimic the native skeletal muscle extracellular matrix (ECM) in vitro. These have the potential to interact with tissues on a cellular and molecular level by imitating mechanical and biochemical cues. Typically, they guide myotube alignment, provide anchorage to contractile myotubes,^[^ ^2^ ^]^ and enhance cellular maturity. In the absence of a supporting scaffold, developing myofibers cultured on rigid tissue culture (TC) surfaces are unstable and detach from their substrate as they contract.^[^ ^3^ ^]^ Both natural and synthetic polymers have been used to fabricate substrates for skeletal muscle tissue engineering. These include encapsulating myofibers in biopolymer hydrogels such as collagen,^[^ ^4^, ^5^ ^]^ fibrin,^[^ ^6^, ^7^ ^]^ gelatin,^[^ ^8^, ^9^ ^]^ and biopolymer mixtures or nanocomposites.^[^ ^10^, ^11^, ^12^ ^]^ Synthetic polymers such as poly(vinyl alcohol), poly(lactic acid), and poly(caprolactone)^[^ ^13^ ^]^ have been fabricated into sheets, porous sponges, and fibers^[^ ^14^, ^15^ ^]^ to guide skeletal muscle organization. Thermoset silicones such as polydimethylsiloxane (PDMS) have been used to cast flexible tendon‐like microstructures, which provide structural supports, such as uniaxial tension across attachment sites.^[^ ^2^ ^]^ These are often used in neuromuscular biosystem applications.^[^ ^16^, ^17^, ^18^ ^]^ Unlike synthetic polymers, most natural biopolymer hydrogels are prone to rapid degradation by the proteases secreted by cells.^[^ ^16^ ^]^ As a result, a fine balance between biopolymer scaffold degradation and the deposition of new ECM by the cells must be achieved to maintain mechanical support and provide the required long‐term myofiber stability. On the other hand, synthetic scaffolds often have high elastic moduli, and lack the appropriate compliance required for supporting skeletal muscle contractility.^[^ ^19^ ^]^ Therefore, novel biomaterials which can be easily fabricated into tissue‐like structures, and which have the biochemical and biophysical properties to support myofiber maturation, integrity, and survival are still highly desired. This includes mechanical properties well‐matched to those of native skeletal muscle, as well as reproducibility and scalability in forming ECM‐like structures with controlled alignment.

Polyurethane (PU) elastomers are widely used in implantable biomedical applications^[^ ^20^ ^]^ because of their excellent hyperelasticity, biocompatibility and fatigue resistance in vivo.^[^ ^21^, ^22^ ^]^ Thermoplastic PU (TPU) and polyurethane‐urea (PUU) copolymers consist of both soft and hard segments, linked by covalent bonds along a linear macromolecular backbone,^[^ ^23^ ^]^ and can be made from a diverse range of raw molecular components. This, and their ease of modification, enables the specification of tissue‐specific properties when used as scaffolds for tissue engineering. In recent years, biobased TPUs made from renewable molecular building blocks have been developed with advanced properties and functions for biomedical applications. This includes highly tunable and responsive mechanical properties, controlled biotic degradation, and low toxic residue formation. TPUs can be easily processed using 3D printing^[^ ^23^ ^]^ and fiber‐spinning technologies such as electrospinning,^[^ ^24^, ^25^ ^]^ enabling the generation of spatially organized scaffolds with ECM‐like microstructure.

Here, a polyester‐based‐PUU elastomer, P(EDS)UU‐POSS, was synthesized by condensation reactions of an ester oligomer diol of ethylene‐diethylene succinate (EDS), a diisocyanate, 4,4′‐methylenebis (cyclohexyl isocyanate) (H_12_MDI) and a diamine chain extender, ethylenediamine, forming aliphatic PUU linkages. EDS is an aliphatic polyester polyol synthesized by polycondensation reaction between renewable succinic acid monomers and ethylene‐ and diethylene‐glycols. The molecules are terminated by tethering polyhedral oligomeric silsesquioxane (POSS) nanocages to the urethane backbone. Siloxanes are well known to be stable in biological settings, resisting oxidation and hydrolysis owing to their unique 3D framework, short bond lengths, and strong intermolecular forces between constituent molecules.

**Figure 1:**
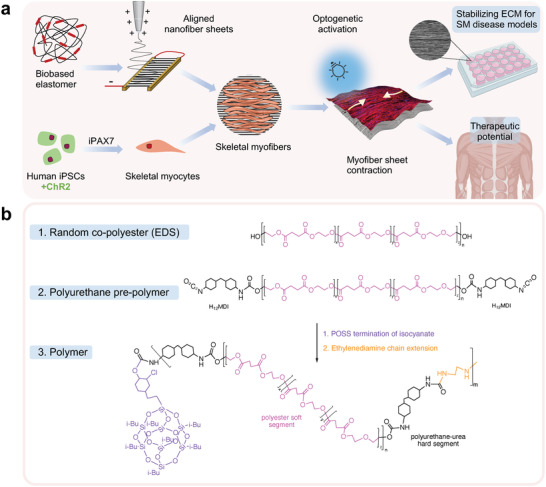
a) Conceptual schematic of biobased elastomer nanofibers for guiding iPSC‐derived differentiation of skeletal myofibers. Aligned nanofibers are obtained by applying an electrostatic field between two aluminum electrodes. Optogenetically controlled (Channelrhodopsin‐2 [ChR2+]) skeletal myocyte progenitors are derived from induced pluripotent stem cells (iPSCs) using inducible Pax7 (iPax7) forward programming. Suspended nanofibers guide the maturation and differentiation of skeletal myofibers and stabilize contractile myofiber sheets. Optogenetic activation enables direct control of quantifiable myofiber contractions. The technology can be used for stabilization and maturation of human iPSC models of skeletal muscle (SM) pathologies in vitro and has potential therapeutic applications. b) Biobased elastomer synthesis steps including molecular structure of polyester polyol EDS, prepolymer polyurethane, and P(EDS)UU‐POSS copolymer nanohybrid.

Taking advantage of its solubility, the P(EDS)UU‐POSS polymer was fabricated into electrospun nanofiber sheets, with the aim of providing an ECM‐like substrate for facilitating skeletal muscle organization in vitro (**Figure** **1**a). These aligned elastomer nanofiber sheets demonstrate superior hyperelastic properties with outstanding compliance matched to that of skeletal muscle tissue, and controllable alignment to guide myotube orientation at a macroscale.

First, the chemical, morphological, and mechanical properties of the polymer and elastomeric nanofiber sheets are investigated. Studies of cell survival, proliferation, and myogenic differentiation of skeletal myoblasts are used to demonstrate that P(EDS)UU‐POSS nanofibers provide a biocompatible ECM‐like substrate for guiding skeletal muscle formation. Finally, when the nanofibers are suspended across custom‐made microwells, a stable and highly aligned elastic sheet‐like structure is formed. This enables the functional behavior of optogenetically controlled human induced pluripotent stem cell (iPSC) derived skeletal myofibers to be assessed. These suspended elastomer nanofibers improve culture longevity and are a promising tool for supporting models of human muscle function and disease.

## 2 Results and Discussion

### 2.1 Synthesis and Characterization of Elastomer Nanofibers

The aliphatic polyester‐based‐PU elastomer (Figure 1b), P(EDS)UU‐POSS, was designed and synthesized as a suitable polymer for use in soft tissue engineering applications. A polyester polyol was first synthesized from renewable monomers by polycondensation reaction, and later formed into a polyester‐based PU polymer by chain extension. Polyester‐PUs made from biobased buildings blocks have been shown to undergo rapid hydrolysis in biological settings.^[^ ^20^ ^]^ However, several studies have reported low cytotoxic effects.^[^ ^26^, ^27^ ^]^ Termination of the polymer chain‐ends with POSS, a silica‐like nanocage, was used to reinforce mechanical and chemical stability. POSS has been shown to shield softer PU segments and repel immediate hydrolytic degradation, as well as to improve hemo‐biocompatibility and antithrombotic effect.^[^ ^28^, ^29^ ^]^ Previous work from our center has shown that POSS‐tethered PUs are more resistant to degradation in vitro and in vivo and sustain cell growth on their surface.^[^ ^30^ ^]^ The use of H_12_MDI for the polyaddition of PU has previously been reported for use in biomedical applications, owing to its low cytotoxicity.^[^ ^31^, ^32^ ^]^ Biobased TPUs have been used for a range of soft tissue engineering applications and have recently been reviewed.^[^ ^33^ ^]^ Several studies^[^ ^34^, ^35^ ^]^ have demonstrated the great potential of biobased TPUs for use in skeletal muscle tissue engineering. Chen et al. showed that the hydrogen bonds which formed between ester, amide, and urea groups of PUU films enhanced the elasticity of the copolymer. The polymer films were shown to support the adhesion, proliferation, and myogenic differentiation of C2C12 myoblasts, but lacked topographical structures to guide myotube orientation.^[^ ^26^ ^]^

The molecular weight and polydispersity of the synthesized EDS polyol and final P(EDS)UU‐POSS elastomer were characterized by gel permeation chromatography (GPC), as listed in **Table** **1**. The resultant chromatogram, and molecular weight distributions are presented in Figure S1, Supporting Information. The molecular weight of the random copolyester polyol had number molecular weight, *M*_n_ = 5237 and a dispersity index, *Ð* = 1.39, indicating relatively uniform chain length. As expected, the final elastomer had a high molecular weight (*M*_w_ = 192 448 g mol^−1^) but a wide dispersity index of 2.9. Multiple factors could contribute to the high polydispersity of final elastomer chain length, including the polydispersity of the polyol, early chain termination by POSS nanocage, the nature of polycondensation, and final chain extension reaction.

**Table 1:** Gel permeation chromatography (GPC) analysis of polyol and polymer samples

	*M*_n_ ^a)^	*M*_W_ ^b)^	*M*_P_ ^c)^	*Ð* ^d)^
EDS Polyol	5237	7265	7391	1.39
P(EDS)UU‐POSS Polymer	65 576	192 448	123 871	2.93

^a)^ Number‐average molecular weight

^b)^ Weight‐average molecular weight

^c)^ Molecular weight of highest peak

^d)^ Dispersion index.

To mimic the spatial and mechanical cues of native extracellular structures, the polymer was processed into thread‐like nanofiber structures using a custom‐built^[^ ^36^, ^37^ ^]^ electrospinning set‐up (Figure 1b). Electrospinning is a simple, reproducible, low‐cost fiber‐forming technology which uses electrostatic forces to generate polymer nanofibers. A variety of nanofiber‐forming techniques, which are driven by other external forces (e.g., gas pressurized, centrifugal) have emerged in the field.^[^ ^38^, ^39^, ^40^ ^]^ Yet, owing to the vast range of spinnable polymers available and its capacity to produce fine nanofibers with highly controlled morphology and properties, electrospinning remains the most versatile and widely used.^[^ ^41^ ^]^ In the present study, electrospinning enabled the precise control over the spatial arrangement of the deposited fibers, generating random or reproducibly aligned nanofiber sheets under a local electrostatic field, by simple manipulation of the collector set‐up. A flat aluminum plate connected to the negative electrode was used to collect randomly oriented fibers, whilst the electrostatic gap collection method was used to collect fibers with uniaxial alignment. Biopolymers like collagen in the extracellular tissues which surround skeletal muscle fibers are essential for guiding morphogenesis.^[^ ^42^ ^]^ As such, unidirectional alignment of the deposited nanofibers is fundamental to mimicking the anisotropic structural organization of the skeletal muscle ECM. Electrospinning enabled the deposition of aligned nanofibers directly onto a format suitable for cell culture, anchoring the fibers in place and maintaining their aligned arrangement (Figure S7a, Supporting Information). As well as this, electrospinning enables the generation of thin ECM‐like sheets with transparency amenable to live‐ and fluorescence‐imaging methods. The structure and properties of the polymer, cast into a solid sheet, as well as the random and aligned nanofiber sheets, were characterized using a range of techniques including Fourier‐transform infrared spectroscopy (FTIR), polarized‐FTIR, scanning electron microscopy (SEM), atomic force microscopy (AFM), mechanical tensile test, and surface contact angle.

**Figure 2:**
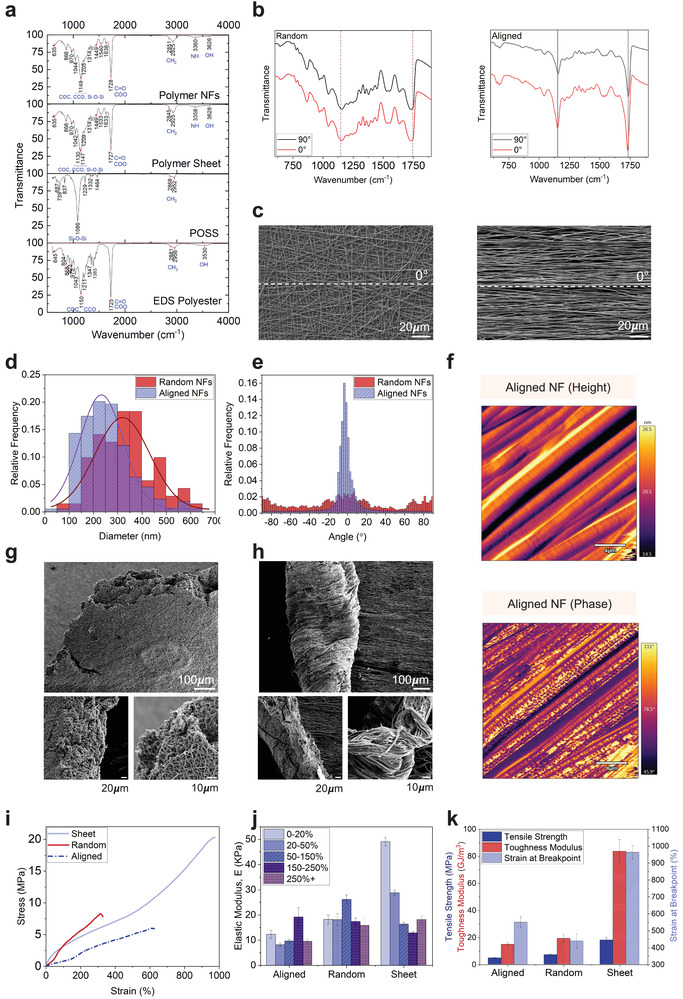
Chemical, morphological, and mechanical characterization of P(EDS)UU‐POSS elastomer nanofibers. a) Fourier transform infrared spectroscopy (FTIR) spectra of EDS polyol, POSS nanostructures, P(EDS)UU‐POSS cast polymer sheets, and nanofiber sheets between 750 and 4000 cm^−1^. b) Polarized FTIR spectra of randomly oriented and uniaxially aligned nanofibers. The infrared beam was polarized parallel (0°) and perpendicular (90°) to the direction of the aligned fibers. To determine the orientation of molecular chains within the nanofibers, the dichroic ratio, DR = *A*_||_/*A*_⊥_ (*A*_||_ = absorbance parallel and *A*_⊥_ absorbance perpendicular) was calculated for individual peaks. c) Scanning electron microscopy (SEM) images of randomly oriented and uniaxially aligned nanofibers. d) Distribution of nanofiber widths quantified from SEM images of randomly oriented and uniaxially aligned nanofibers. Unpaired *t*‐test, *p* < 0.0001. e) The radial integration of power spectrum as a function of angle by fast Fourier transform (FFT) analysis of SEM images for measurement of fiber alignment. Gaussian fit, *R* ^2^ = 0.9711, Two tailed, unpaired *t*‐test, *p* < 0.0001. f) Atomic force microscopy (AFM) images of height and phase of aligned nanofiber structures. g) SEM images of the aligned and h) random nanofiber sheets post tensile fracture. i) Stress–strain curves, j) elastic modulus over a range of strain groups (0–50%, 50–150%, 150–250%, 250%+), and k) tensile strength, toughness modulus, and strain at break for P(EDS)UU‐POSS polymer cast solid sheets, electrospun random nanofiber sheets, and uniaxially aligned nanofiber sheets. Full details of *n* numbers, statistical methods, and *p* values are detailed in Table S4, Supporting Information.

The characteristic molecular functional groups of the POSS nanocage as well as the EDS polyol and P(EDS)UU‐POSS polymer were confirmed by measuring the ATR‐FTIR spectrum (**Figure** **2**a). The absorption peaks detected in response to specific IR wavenumbers correspond to characteristic vibrations of specific chemical bonds present in each sample. In both solid and nanofiber polymer samples, the peaks at 3358 (solid sheet) and 3360 cm^−1^ (nanofiber) correspond to the stretch of the hydrogen‐bonded secondary amine, −NH,^[^ ^43^, ^44^, ^45^ ^]^ which form the urea and urethane groups of the polymer. As expected, those peaks were not present in the POSS or polyol samples. Broad absorption peaks in the similar wavenumber range between 3530 and 3628 cm^−1^ observed in the polyol were assigned to the stretching of its terminal —OH groups. The peaks at 2845–2956 cm^−1^ correspond to symmetrical and asymmetrical —CH_2_ stretching.^[^ ^43^ ^]^ The absence of the band at 2260 cm^−1^ proves the completion of the reactions between —NCO and —OH and absence of free —NCO groups.^[^ ^46^ ^]^ In polymer samples, the typical peak assigned to the stretching vibration of the carbonyl bonds, —C=O,^[^ ^46^, ^47^, ^48^ ^]^ which form part of the urethane (—NCO—), urea (—CO(NH)_2_—), and ester (—COO—) groups, are visible at 1725–1728 cm^−1^,^[^ ^48^ ^]^ likewise the carbonyl bond in the ester groups of the polyol. The clear peak at 1086 cm^−1^ in the POSS sample was assigned to —Si—O—Si— vibrations from the silsesquioxane groups.^[^ ^49^, ^50^ ^]^ The broader, less strong peaks around 1100 cm^−1^ in the polymer samples could be assigned to a combination of the —Si—O—Si— vibrations of the silsesquioxane groups, as well as the aliphatic ether —C—O—C— stretch and ester O—C—C stretch.^[^ ^46^ ^]^

The FTIR set‐up was then adapted to incorporate a polarizing filter. Polarized FTIR has been used previously as a simple and effective method to investigate chemical bond orientation of polymer chains within macroscopically aligned nanofibers.^[^ ^51^, ^52^, ^53^, ^54^ ^]^ It is widely recognized that the degree of polymer chain orientation can be estimated from the dichroic ratio, DR = *A*_||_/*A*_⊥_, where *A*_||_ is the absorbance for IR light polarized parallel to the fiber axis, and *A*_⊥_, is the absorbance for IR light polarized perpendicular to the fiber axis. This relationship was first defined in the work of Fraser^[^ ^55^ ^]^ in 1958 and considers the angle between the transition dipole moment direction and the molecular axis to be important in defining molecular orientation within a single polymer nanofiber.^[^ ^56^ ^]^ Theoretically, in a randomly oriented sample, the DR = 1, whilst in uniaxially oriented samples the DR = ∞. In this study, the polarized FTIR spectrum for macroscopically randomly oriented and aligned nanofiber sheets was analyzed. The calculated DR and estimated α interval (Equations S1 and S2, Supporting Information) for the characteristic peaks was calculated and is presented in Table S2, Supporting Information. For randomly oriented samples, the DR for all measured peaks was close to 1, whilst for aligned nanofiber sheets, the relative intensity of the peaks was much larger when the IR light was polarized parallel to the direction of macroscopic fiber alignment, resulting in DR > 1. The relative absorption of two distinct peaks between 625 and 1875 cm^−1^ are highlighted in Figure 2b. In the case of the aligned nanofibers, the peak at ≈1140 cm^−1^ is likely assigned to —C—O—C— stretching vibration which is stronger in parallel to the nanofiber orientation. This indicates that within the polymer main chains, the chain segments consisting of —C—O—C— bond at least, are oriented along the nanofiber axis. The most pronounced band ≈1715 cm^−1^ corresponding to the —C=O stretching vibration follows the same trend parallel to the fiber orientation, which is unexpected as the —C=O stretching movement should, in theory, be perpendicular to the main chain. It is noted that the —C=O bond, together with its belonging —COO— or —NCO—, —CO(NH)_2_— groups are polar, which implies they have readily oriented themselves along the electric field under which the nanofibers are generated and stretched during electrospinning. Clearly, the forces involved in forming the nanofibers result in electric field‐induced polar group orientation in the molecular chains.^[^ ^57^, ^58^ ^]^ As a result, some polymer chain segments are oriented along the fiber long axis, while others may remain tangled together, with all the polar groups well aligned along the fiber direction.

It has also been widely reported that higher molecular alignment is associated with small fiber diameters of electrospun fibers.^[^ ^59^, ^60^ ^]^ The diameters of individual nanofibers with random and aligned orientation were quantified from SEM images (Figure 2c). Figure 2d illustrates the distribution of diameters measured. A Gaussian fit was applied to the distribution which indicated that the aligned nanofibers were significantly smaller in diameter (253.7 ± 103.6 nm) than randomly oriented fibers (332.4 ± 116.1 nm) under the same electrospinning conditions. Macroscopic alignment and distribution of the nanofibers was further analyzed by fast Fourier transform analysis (FFT), using the Directionality plugin in FIJI.^[^ ^61^ ^]^ A clear preferred orientation (−2.6 ± 4.4°) was obtained from the angular distribution function (Figure 2e), indicated by the intensity peak close to 0°, which is parallel to the fiber orientation in the SEM image. In contrast, no preferred orientation (1.23 ± 199.3°) was observed in the randomly oriented fibers.

Understanding the surface properties of a material is important in identifying new materials for tissue engineering. Topographical cues can facilitate cellular adhesion and differentiation.^[^ ^62^ ^]^ AFM images revealed further information about the surface topography, and phase structure along the length of the aligned nanofibers. Images of the amplitude of the AFM cantilever oscillations (Figure 2f) revealed that the aligned nanofibers had a smooth topological surface with height variation within several nanometers, despite the observation of ordered ridge‐like structures aligned along the fiber axis, as well as less prominent but visible indents perpendicular to the fiber axis. Phase images of these regions revealed heterogeneous surface stiffness at nanoscale distributed along the fibers. This could be attributed to the self‐assembled nanophase separation of soft and hard segments of P(EDS)UU‐POSS copolymer, which were also visible in phase images of the flat cast polymer sheet (Figure S2, Supporting Information).

The stress–strain behavior of the P(EDS)UU‐POSS cast solid sheets and electrospun nanofiber sheets was analyzed using static tensile loading. SEM images of the post‐fracture samples are shown in Figures 2g (random) and 2h (aligned). The fractured edge of the random nanofibers had a layered structure with a relatively blunt, porous fibrous network morphology. In contrast, the aligned fibers formed a rough and uneven fracture surface with crimping fibrous bundles, due to the elongated fibers’ pull‐out failure when fractured. Figure 2i shows the stress–strain curves obtained from applying a constant 5 kN load at 2 mm s^−1^ to dumbbell‐shaped samples of the polymer sheet, random nanofibers, and aligned nanofibers. The remarkable ultimate strain of the polymer sheet between 900% and 1000% demonstrates the exceptional hyperelasticity of the polymer. The samples exhibited different regimes of hyperelastic behaviors. These correspond to low, moderate, and high chain extension conformations during stretching, with a range of elastic moduli, *E*, obtained (Figure 2j) over a range of strain groups (0–20%, 20–50%, 50–150%, 150–250% and 250%+). As with most biological tissues, skeletal muscle has been shown to exhibit nonlinearly elastic behavior.^[^ ^63^ ^]^ The stiffness (elastic modulus) of native skeletal muscle is reported to range from ≈11 to 50 kPa.^[^ ^63^, ^64^, ^65^, ^66^, ^67^ ^]^ Regular TC polystyrene plastic is six orders of magnitude stiffer, at ≈1 × 10^7^ kPa. Substrate stiffness has been shown to influence myoblast differentiation. Engler et al.^[^ ^64^ ^]^ showed that the optimal stiffness for proper myotube formation in C2C12 myoblasts matched that of native skeletal muscle, at 12 kPa. Myotubes with too low (<5 kPa) or too high (>17 kPa) stiffness did not form myosin striations. **Table** **2** shows the experimentally obtained average (across all strain groups) elastic modulus of the cast solid polymer sheet (25.1 ± 1.0 kPa), random nanofiber (19.2 ± 1.9 kPa), and aligned nanofiber sheets (11.8 ± 1.2 kPa). Whilst all three forms of elastomer sheets are considerably lower than regular polystyrene TC plastic, the stiffness of the aligned nanofibers appears well matched to that of C2C12 myofiber growth as aforementioned.

**Table 2:** Mean modulus of elasticity, E (KPa), for native skeletal muscle, TC plastic and experimentally obtained P(EDS)UU‐POSS polymer cast solid sheet, random nanofiber sheets, and uniaxially aligned nanofiber sheets

	Native SM^c)^	Aligned	Random	Sheet	TC^d)^ Plastic
Mean^a)^ E^b)^ [kPa]	≈11–50	11.8 ± 1.2	19.2 ± 1.9	25.1 ± 1.0	1 × 10^7^

^a)^ Mean across all strain groups

^b)^ Elastic modulus

^c)^ Skeletal muscle

^d)^ Tissue culture.

Figure 2k shows the tensile strength, toughness modulus, and strain at breakpoint for the three forms of elastomer sheet. As expected, all parameters were found to be significantly greater in the polymer solid sheet when compared with aligned or random nanofibers with porous structure (Tukey's Multiple comparison test, *p* < 0.0001). Moreover, tensile strength was significantly greater in the random nanofibers consisting of more entanglements and fiber‐fiber adhesion than aligned nanofibers (*p* = 0.0428), whilst strain at break was significantly higher in the aligned nanofibers than random nanofibers (*p* = 0.0272). This suggested that random nanofibers could withstand higher stress than aligned nanofibers; however, aligned nanofibers could withstand higher strain and therefore stretch more before fracture. As a result, the hyperelasticity of the aligned electrospun nanofibers is envisaged to be a promising scaffold for achieving synergistic displacement upon myofiber contraction.

### 2.2 Cellular Interaction with Elastomer Nanofibers

To assess the biocompatibility of P(EDS)UU‐POSS nanofibers, several cell culture assays were carried out using C2C12 myoblast cells. The viability of C2C12 myoblasts grown on glass coverslips (control), and coverslips coated with either randomly oriented or aligned nanofibers was quantified from immunocytochemistry/immunofluorescent (ICC/IF) images (Figure S3a, Supporting Information) of Live/Dead (calcein‐AM/ethidium homodimer) staining over 10 days. The percentage of viable cells (Figure S3b, Supporting Information) was maintained close to 90% in all conditions, with no significant differences (Table S4, Supporting Information) in viability observed between each condition or within each condition over the 10‐day culture period. This demonstrates that the P(EDS)UU‐POSS elastomer is nontoxic and capable of promoting the growth of myoblasts and myofiber‐like cells.

The metabolic activity of the C2C12 cells grown on random and aligned nanofibers was quantified using the PrestoBlue assay (Figure S4, Supporting Information) over a 10‐day differentiation period. When compared with cells grown on glass, no significant differences (Table S4, Supporting Information) in metabolic activity were seen on days 1 and 5. Interestingly, on day 10, cells grown on nanofibers (both random and aligned) maintained similarly high levels of metabolic activity as on day 5, whereas cells grown on the glass control had significantly lower levels. Despite not being a direct indicator, the PrestoBlue assay for cell metabolic activity is often inferred as an indicator of cell viability. However, cellular metabolism has been shown to decrease in differentiating cells,^[^ ^68^ ^]^ and therefore the maintained levels of metabolic activity shown here are a positive indication that the cells retained high activity levels when attached to the underlying nanofiber substrate. It is clear the nanofibers provide topographical attachment sites which promote culture longevity and, in turn, improved myogenic differentiation potential.

**Figure 3:**
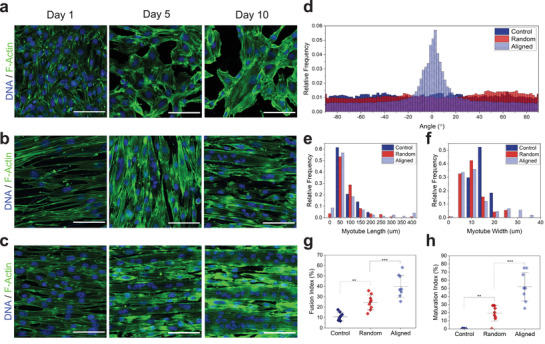
Myotube morphology and differentiation on elastomer nanofibers. a−c) Immunocytochemistry (ICC) images of C2C12 myoblasts stained for F‐actin (green) and DNA (blue) grown on glass coverslips (control) (a) and b,c) on coverslips coated with randomly oriented (b) and uniaxially aligned (c) P(EDS)UU‐POSS electrospun nanofibers, on days 1, 5, and 10. d) Myotube alignment quantification using fast Fourier transform (FFT) analysis to determine directionality of myoblasts grown on each substrate condition. e) Frequency distribution of myotube lengths and f) widths for myoblasts grown on each substrate condition on day 10. g) Myotube fusion index (MFI) and h) myotube maturation index (MMI) for myoblasts grown on each substrate condition on day 10. Scale bars: 100 µm. **** = *p* value < 0.0001.

Physical, topographical, and mechanical properties have been shown to enhance cell adhesion, proliferation, and differentiation potential of different musculoskeletal cell types.^[^ ^69^ ^]^ To assess how the electrospun nanofibers guide myoblast orientation and contribute to myofiber maturation, the orientation and morphology of C2C12 myoblasts were analyzed. ICC images of cells stained for F‐Actin (Phalloidin) and DNA (DAPI) showed visible morphological differences in the myotubes which formed on glass coverslips (**Figure** **3**a), and on coverslips coated with randomly oriented (Figure 3b) and aligned nanofibers (Figure 3c). The orientation of phalloidin‐stained structures in each condition was analyzed by FFT in the FIJI software package on day 10 (Figure 3d). Consistent with what is observed in the ICC images, the sharp peak observed at 0° (Horizontal) in the aligned nanofiber group indicated a preferred orientation of phalloidin‐stained actin cytoskeleton structures along the aligned axis of the nanofiber substrate. In contrast, phalloidin staining of C2C12 myoblasts on randomly oriented nanofibers and on glass coverslip controls had no preferred orientation, with cells not aligning and forming a peak in any single direction.

As well as the differences in myotube orientation, distinctly different distributions of measured lengths and widths of myotubes which formed in each condition are shown in Figures 3e and 3f, respectively. Cells grown without nanofibers formed shorter (50–200 µm) and thinner (10–20 µm) myotubes. In contrast, culturing the cells on a nanofiber substrate facilitated the formation of longer and thicker myotubes, with both random and aligned nanofibers forming myotubes up to 400µm in length. Unidirectional alignment of the nanofibers had a notable effect on myotube width, with myotubes up to ≈35 µm in width forming on aligned samples only.

Two common measures^[^ ^70^ ^]^ of myotube maturation were used as measures of differentiation potential on nanofiber‐coated coverslips. Overall, Myotube fusion index (MFI), which is the fraction of myotubes containing two or more nuclei, was found to be significantly higher in both nanofiber groups (*p*_random_ = 0.0025, *p*_aligned_ < 0.0001) when compared with the glass control (Figure 3g). MFI was also significantly (*p* = 0.0008) higher on aligned nanofibers (mean_MFI_ = 39.91 ± 9.97%) than on random nanofibers (mean_MFI_ = 24.58 ± 6.78%). Similarly, myotube maturation index (MMI), which is defined as the fraction of myotubes containing five or more nuclei, was significantly higher in both nanofiber groups (*p*_random_ = 0.0043 and *p*_aligned_ < 0.0001, respectively) compared to the control, and significantly higher (*p* < 0.0001) on aligned (mean_MMI_ = 51.98 ± 16.49%) nanofibers than random (mean_MMI_ = 19.35 ± 9.08%) nanofibers (Figure 3h).

These data suggest that on aligned nanofibers, topographical guidance directs myotube orientation, and in turn facilitates the recruitment of larger numbers of myoblasts into each myotube, leading to the formation of larger, more mature myotubes. This, combined with the successful biocompatibility and metabolic activity tests, suggests that P(EDS)UU‐POSS elastomer nanofibers provide an excellent substrate for further investigation for skeletal muscle tissue engineering.

### 2.3 Assembly of Suspended Nanofiber Sheets

Collecting nanofibers directly onto glass coverslips and placing them inside multiwell microplates is a common and widely used method for seeding cells and assessing cellular interaction. However, using this method, the nanofibers are immobilized and cannot deform freely when they are collected on and attach to the glass coverslip. To take advantage of the hyperelastic properties of the P(EDS)UU‐POSS nanofibers, a method to assemble suspended nanofiber sheets (SNFs) in a standard TC format was developed. The necessity to assemble the nanofibers in this form is supported by the work of Sheets et al.^[^ ^71^ ^]^ They described suspended nanofibers as 1D beams of uniform material stiffness but with varying structural stiffness along their length. They showed that the location of cells along a single suspended fiber could affect specific cell behaviors such as cell spreading, migration, and cytoskeletal arrangement.^[^ ^71^ ^]^ In contrast, cells grown on flat TC surfaces experienced material stiffness which was uniformly distributed and constant across their focal adhesions.

**Figure 4:**
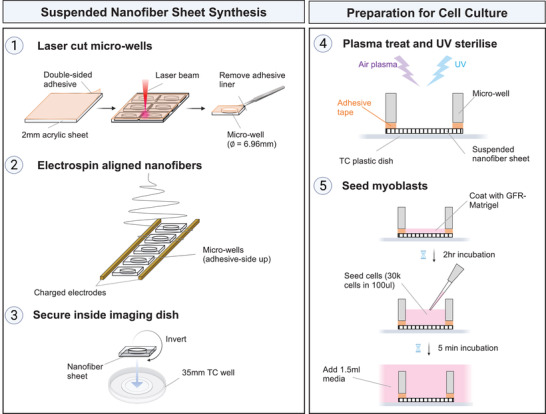
Assembly of uniaxially aligned suspended nanofiber sheets (SNFs). 1) Microwells with 6.96 mm diameter were cut from 2 mm thick acrylic sheet coated with double sided adhesive tape. 2) Individual microwells were placed, adhesive‐side up, between two charged electrodes. Aligned nanofibers were electrospun across the microwells. 3) The microwells, with nanofibers suspended across one side, were inverted, and secured to the base of a 35 mm tissue culture dish. The adhesive tape served to anchor the nanofibers to the edges of the microwell, and to attach the well to the dish. 4) The assembled wells were air plasma treated and UV sterilized to remove surface contamination. 5) The SNFs were precoated with GFR‐Matrigel before cell seeding. Myogenic progenitors were seeded directly into the central well in a concentrated (30 000 cells/100 μL) volume of media. After 5 min, or once the cells had visibly settled to the bottom of the well, the tissue culture dish was topped up with 1.5 mL media.

The schematic diagram shown in **Figure** **4** demonstrates the assembly process in which: 1) microwells were laser‐cut from an acrylic sheet, 2) aligned nanofibers were electrospun across the microwells, and 3) the microwells, with nanofibers suspended across the bottom, were secured to the base of a 35 mm TC dish. The adhesive tape served to anchor both the nanofibers to the edges of the microwell, whilst allowing attachment of the well to the dish. Next the SNFs were prepared for cell culture. 4) Air plasma treatment was used to functionalize the nanofiber surface and increase hydrophilicity (Figure S5, Supporting Information), and UV sterilization was used to prevent culture contamination. 5) The nanofibers were coated with growth factor reduced (GFR)‐Matrigel prior to cell seeding. Cells were then seeded directly into the central well (30 000 cells in 100 µL) and allowed to settle on the SNFs for 5 min. The wells were then topped up with media and maintained at 37 °C, 5% CO_2_.

The effect of plasma surface modification on cell–substrate interactions has been widely studied.^[^ ^72^, ^73^ ^]^ Synthetic polymers used in biomedical applications often have low surface energy and hydrophobic properties. Plasma modification is a solvent‐free method used to alter the surface chemistry of a polymer, without changing its’ bulk properties. The introduction of surface functional groups, such as —OH, —COOH, NH_2_, and radicals, functionalizes the polymer surface,^[^ ^74^ ^]^ altering surface properties such as wettability, polarity, protein adsorption and, in doing so, improving cellular behavior.^[^ ^75^ ^]^ Lie et al. demonstrate the introduction of oxygen‐containing polar groups to PLLA nanofibers using plasma treatment. This resulted in a significant increase in hydrophilicity and subsequent adhesion and spreading of mesenchymal stem cells on the nanofiber surface.^[^ ^76^ ^]^ Similarly, in the present study, air plasma treatment introduces hydrophilic functional groups and radicals to the nanofiber surface, increasing surface wettability and significantly reducing the water contact angle (Figure S5, Supporting Information). This facilitates the attachment of ECM‐proteins such as laminin during Matrigel‐coating.^[^ ^77^ ^]^

When C2C12 myoblasts were cultured on SNFs, the resulting myofibers had visibly organized filamentous actin and sarcomere‐like periodic striations along their length (Figure S6, Supporting Information), suggestive of sarcomere formation. This was not observed in C2C12 cells previously grown on nanofiber‐coated coverslips (Figure 3). This supports the hypothesis that nanofibers collected directly onto coverslips are immobilized by the rigid surface and cannot displace in response to the force generated during contraction of the overlying myofibers. The results also agree with a study by Serena et al. in which they show that substrate stiffness plays a role in sarcomere organization. They found that human myotubes only formed organized sarcomeric myosin and α‐actinin structures when grown on soft (*E* ≈ 15 kPa) micropatterned hydrogels, whereas myotubes grown on glass did not form structured sarcomeres.^[^ ^78^ ^]^

In line with the presence of ordered sarcomeres, the C2C12 myofibers we observed on SNFs exhibited spontaneous but continuous contractions along their length. Myofibers which formed on a regular TC plastic control well exhibited only sporadic twitching in some regions. When this dynamic movement was quantified with particle image velocimetry (PIV),^[^ ^16^ ^]^ both the magnitude and frequency of contraction were much greater in C2C12 myofibers which formed on SNFs than on the control wells (Figure S6, Supporting Information). These promising observations suggest that SNFs improve the organization and performance of contractile skeletal myofibers in functional in vitro studies.

### 2.4 Suspended Nanofibers Sheets Support Contractile Function in Human‐iPSC‐Derived Myofibers

One of the key challenges in skeletal muscle tissue engineering is the ability to stabilize contractile myofibers and prevent premature collapse. To further test how the P(EDS)UU‐POSS elastomer nanofibers could support contractile myofibers in vitro, we generated human‐iPSC‐derived myofibers whose contractile function could be controlled with optogenetics. Myogenic progenitor cells were derived from a human iPSC line and further differentiated into myofibers.

**Figure 5:**
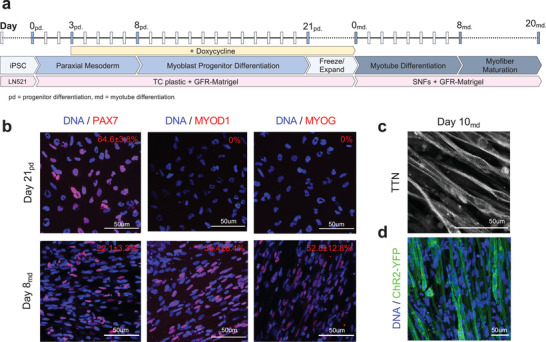
Generation of optogenetically controlled human‐iPSC‐derived myofibers. a) Differentiation timeline from iPSCs to myoblast progenitors (progenitor differentiation [pd]; day 0_pd_–21_pd_), from progenitors to myotubes (myotube differentiation [md]; day 0_md_–8_md_), and for myotube maturation into myofibers (day 8_md_–20_md_). b) Immunocytochemistry (ICC) images and quantification of myoblast progenitors (day 21_pd_) and myotubes (day 8_md_) expressing PAX7, MYOD1, and MYOG. c) Immunocytochemistry (ICC) image of day 10_md_ myotubes with striated sarcomeric Titin (TTN) expression. d) Expression and quantification of transgenic ChR2‐YFP in day 8_md_ myotubes.

A previously reported^[^ ^79^ ^]^ forward programming approach was used for the differentiation, with a doxycycline‐inducible PAX7 myogenic determinant inserted by gene targeting into the CLYBL safe‐harbor locus^[^ ^80^ ^]^ in human iPSCs. The differentiation timeline is shown in **Figure** **5**a. After a 21‐day progenitor differentiation (pd) (0_pd_–21_pd_) period, 60–70% of cells expressed PAX7 (Figure 5b). After a further 8‐day myotube differentiation (md) (0_md_–8_md_) period, PAX7 protein was detected in 20–30% of all cells, whilst the downstream muscle transcription factors MYOD1 and MYOG were expressed in >50% of cells. After 10 days of md, the iPSC‐myoblasts had fused to form myofibers which expressed Titin (TTN) (Figure 5c) in a periodic striated sarcomere pattern. To enable optogenetic activation, the light‐gated ion channel Channelrhodopsin‐2 (ChR2)^[^ ^81^ ^]^ was stably integrated into the genome of the iPAX7 human iPSC line by PiggyBac‐mediated transposition. The iPAX7/ChR2‐YFP iPSC cell line was then expanded and differentiated into myofibers which homogeneously expressed ChR2‐YFP (Figure 5d). ChR2 is the most commonly used optogenetic actuator, and our group has previously characterized its electrophysiological properties in embryonic‐stem‐cell‐derived MNs.^[^ ^16^, ^82^ ^]^ In this study, the ChR2 was stably integrated directly into the iPSCs which would later form the myofibers, enabling direct optical activation^[^ ^83^ ^]^ of the formed myofibers. This bypasses the requirement for more complex neuro‐muscular coculture systems, which are not necessary for assessing initial cell–substrate interactions.

**Figure 6:**
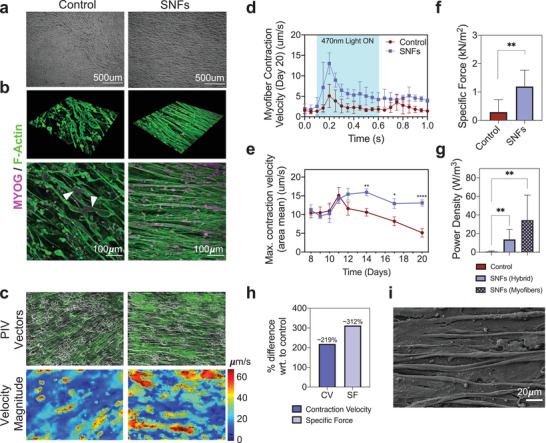
Suspended nanofibers sheets stabilize optogenetically controlled contractions in human‐iPSC‐derived myofibers. a) Bright‐field images of human‐iPSC‐derived myofibers cultured on tissue culture (TC) plastic (control) and on suspended nanofibers (SNFs). b) 3D and 2D immunocytochemistry (ICC) images of day 14 cultures. Cells are stained for F‐Actin and Myogenin (MYOG). c) Particle image velocimetry (PIV) vector plots and velocity magnitude heat map of optogenetically controlled contractions at contraction peak. d) Myofiber contraction velocity (CV) trace obtained from PIV videos of myofiber contractions on control and SNFs on day 20. The blue region in the graph indicates the 0.5 s time window where the 470 nm light is on. Mean ± standard deviation (SD). e) Area mean peak contraction velocity (µm s^−1^) on control wells and on SNFs over days 8_md_–20_md_. f) Specific force (SF, kN m^−2^) and g) power density (PD, W m^−3^) on day 20. h) Percentage difference between CV and SF with respect to (wrt.) control on day 20. i) Scanning electron microscopy (SEM) image of myofibers cultured on SNFs. Statistical analyses performed are detailed in Table S4, Supporting Information. **** = *p* value < 0.0001.

After the 21‐day pd period, the iPAX7/ChR2‐YFP human‐iPSC‐derived myogenic progenitors were seeded onto SNFs and further differentiated into myofibers. Consistent with what we observed in C2C12 cultures, the progenitors became oriented along the axis of the nanofibers, before further differentiating and fusing to form a globally aligned myofiber culture (**Figure** **6**a) over a 20‐day culture period. The cultures were stained for F‐Actin and the myogenic transcription factor Myogenin, to visualize the myofiber arrangement (Figure 6b). On SNFs, the cultures maintained their density and appeared to form a sheet‐like structure for the duration of the 20‐day culture period. In contrast, the appearance of collapsed or detached myofibers was clearly visible on the regular TC control wells. On days 8_md_–20_md_, each myofiber culture was activated with a single 0.5s blue (470nm) light pulse. Upon stimulation, the myofibers rapidly contracted and then relaxed to a prestimulation state. Videos of the contractions were recorded with brightfield microscopy (Videos S1 and S2, Supporting Information). The mean (of the field of view [FOV]) contraction velocity of the myofiber sheet was quantified using PIV analysis. The displacements were represented by an array of vectors (Figure 6c and Figure S8a, Supporting Information) which, on the SNFs, were oriented along the nanofiber axis (0° and 180°), and on the control wells, were oriented in all directions. The magnitude of the velocities across a single FOV at the contraction peak is represented by the heat maps. Figure 6d illustrates the contraction velocity time‐course in response to the 0.5 s blue LED pulse on day 20. Contractions were measured on days 8, 10, 12, 14, 17, and 20, and the peak contraction velocity recorded (Figure 6e). Between days 8 and 10, the peak contraction velocity was maintained at ≈10 µm s^−1^ in both conditions, which increased to ≈15 µm s^−1^ on day 11. From day 12 onwards, the maximum contraction velocity decreased significantly in the control group, consistent with the appearance of collapsed or detached myofibers visible in the cultures. This collapse process is clearly visible in Video S1, Supporting Information, in which single myofibers contract and detach from the TC plastic. In contrast, the myofibers on SNFs maintained a significantly higher contraction velocity (>200% higher on day 20), with myofibers contracting and relaxing whilst maintaining their prestimulation arrangement.

The force (N) and power density (W m^−3^) of the contracting myofiber constructs was estimated from the PIV output data using a traction force microscopy (TFM)‐inspired approach. TFM is typically used to measure the magnitude and the direction of forces (tractions) produced by adherent cells on a substrate of known stiffness. Teo et al.^[^ ^84^ ^]^ report a simple method to use PIVLab velocity output data for TFM. To obtain force measurements (specific force, kN m^−2^) comparable to other studies, we used Hooke's laws to calculate an estimate of the stress vectors and subsequent force vectors in *X* and *Y* directions in the contracting myofiber–nanofiber sheet. As expected, the data obtained correlated with the significant differences in contraction velocity observed. Overall specific force was significantly higher on SNFs (1.19 ± 0.5 kN m^−2^) than on regular TC plastic controls (0.28 ± 0.4 kN m^−2^) (Figure 6f). Interestingly, the highest contribution to this difference was in the *X* direction (Figure S8b, Supporting Information), which corresponds to the longitudinal axis of the aligned nanofibers. Differences in specific force in the *Y* direction were not significant between SNFs and controls. This supports our findings that concurrent alignment between the nanofibers and nanofibers exist, and elucidates that the contractile forces are transmitted along this axis. The power density (W m^−3^), which considers the volume of the myofiber–nanofiber hybrid construct, also followed this trend (Figure 6g). In addition, to determine the power density of the engineered skeletal muscle itself, the power density was also calculated by considering the volume of the myofiber layer only. Figure 6h illustrates the percentage difference between the contraction velocity (≈216%) and specific force (≈282%) of the SNF‐myofiber hybrid with respect to the control wells.

The end‐point cultures were also visualized by SEM (Figure 6i and Figure S7b, Supporting Information) revealing visible connections between the myofibers and the underlying elastomer nanofibers.

The results from this functional study of myofiber contraction indicate that physical topographical cues not only guide myotube alignment, but also aid attachment of the cells to the underlying substrate and aid force transmission. We propose that functionalizing the nanofiber surface by plasma treatment enhanced the immobilization of biologically active ligands present in the culture media and Matrigel coating on the fiber surface, prior to cell seeding, which improved the adhesion between the fiber substrate and the cells. Amongst other proteins and factors, Matrigel is rich in ECM proteins laminin (≈60%) and collagen IV (≈30%). Laminins regulate cell adhesion and migration and transmit forces from the ECM through integrins and focal adhesions to the actin cytoskeleton.^[^ ^85^, ^86^ ^]^ Since these surface functional proteins were also present in the control samples, the geometry, topographical arrangement, and mechanical properties of the nanofibers themselves clearly aid attachment, myotube formation, and force transmission in the cultures as they mature (Figure 6 and Figure S8, Supporting Information). The fibrous structure of the nanofibers mimics the fibrous proteins of the native ECM, providing similar motifs for cell adhesion and myofiber growth. Increased surface area afforded by nanostructured substrates has been shown to substantially increase the size and numbers of focal adhesions.^[^ ^87^ ^]^ In addition, the alignment and matched elasticity of the nanofiber sheet profoundly enhance force transmission between the cells and the artificial ECM. Cells attached to suspended nanofibers form strong focal adhesions along the fibers, particularly at the nanofiber poles, which act to engender contractile forces without frictional resistance.^[^ ^88^, ^89^ ^]^ Owing to the matched mechanical properties and strong adhesion between the myofibers and the nanofibers, their synergistic contraction in response to light stimulation, and subsequent stability, have been demonstrated.

Several novel biomaterial scaffolds and creative biofabricated platforms have been developed to support in vitro skeletal muscle tissues with the desired physiological and functional complexity. These biomimetic strategies not only drive the development of regenerative therapies to treat volumetric muscle loss, but also provide a representative platform for the study of human skeletal muscle pathologies such as Duchenne muscular dystrophy, and muscle aging. As well as obtaining the relevant cell types, the in vitro microenvironment must be sufficiently stable to model slow pathological processes. Existing platforms range from microfluidic chambers^[^ ^90^ ^]^ and flexible PDMS posts,^[^ ^91^ ^]^ to neuromuscular organoids^[^ ^92^ ^]^ and 3D printed cell‐laden hydrogels^[^ ^93^ ^]^ and scaffolds. Often, these methods take inspiration from classic tissue engineering approaches in which cells are embedded and cultured within an exogenous 3D matrix made from biopolymers native to the skeletal muscle ECM. Several groups have successfully formed suspended “myobundle” muscle constructs by encapsulating cells in biopolymer gels such as collagen and fibrin, and subsequently holding them under tension between two uniaxial attachment sites.^[^ ^18^, ^94^ ^]^ These 3D myobundle systems exhibit aligned native muscle architecture,^[^ ^95^ ^]^ enable functional contractile force measurement, and can mimic clinical responses of skeletal muscle to drugs.^[^ ^96^ ^]^ Whilst excellent progress has been made in this type of 3D system, their weaknesses are twofold.

First, biopolymers are rapidly remodeled and degraded by the embedded cells. While the cells themselves are expected to synthesize and secrete endogenous ECM matrix proteins, this process is relatively slow,^[^ ^86^ ^]^ particularly where the myoblasts are not supported by high ECM‐depositing cells such as myo‐fibroblasts.^[^ ^97^ ^]^ Without stabilizing agents, biopolymers like fibrin are significantly degraded by endothelial cells within 7 days.^[^ ^98^ ^]^ Myofiber constructs made from rat skeletal myoblasts embedded within collagen gels are shown to rupture within 5–7 days of culture. While mixtures of collagen, fibrinogen, and Matrigel hydrogels were shown to prolong this and aid force transmission,^[^ ^86^ ^]^ compaction of the gels by the cells was still apparent. While biopolymers provide important biochemical cues for cell attachment and differentiation, culture maturity is limited by their inherent degradation properties. As such, use of synthetic scaffolding materials functionalized with biopolymers is an attractive alternative. Often, these systems can successfully provide force and other biochemical‐based read‐outs, but additional post‐processing steps are required for image‐based screening. On top of this, most published 3D approaches are less compatible with high‐throughput screening and automation.^[^ ^99^ ^]^ While some progress has been made in scaling‐up these technologies,^[^ ^100^ ^]^ most existing platforms involve challenging, cumbersome multistep fabrication, and assembly processes. This limits the widespread use of such systems for disease modeling and drug screening.

With these limitations in mind, we developed a nanofiber‐based suspended ECM system made from a new synthetic elastomer which has mechanical properties matched to that of skeletal muscle. The elastomer nanofibers support adhesion, alignment, and differentiation of iPSC‐derived skeletal myofibers over a 20‐day period. Finally, we estimate the specific force and power density exerted by the iPSC‐derived nanofiber‐myofiber sheets, using a standard microscopy set‐up and simple computational PIV analysis pipeline. We demonstrate that the myofibers are stable after optogenetic activation, have a power density of ≈35 W m^−3^, and exhibit a significantly higher contraction velocity (>200%) and specific force (>280%), than myofibers grown on traditional TC plastic. While our study does not permit the direct measurement of contractile forces exerted by the skeletal myofiber constructs, this computational approach provides a simple noninvasive microscopy‐based method to estimate the contraction force and power density exerted by the cells. The calculated specific force is within the range of values obtained from existing systems^[^ ^18^, ^79^, ^86^, ^95^, ^100^, ^101^, ^102^, ^103^, ^104^, ^105^, ^106^, ^107^, ^108^, ^109^, ^110^, ^111^, ^112^, ^113^, ^114^, ^115^, ^116^, ^117^, ^118^ ^]^ (Table S3, Supporting Information) used to stabilize and measure contractile force of tissue‐engineered skeletal muscle in the literature. While the accuracy of the estimation made needs further experimental validation, this computational pipeline is a promising tool which can be used routinely alongside standard biochemical assays to assess functional responses of tissue‐engineered myofiber constructs to experimental stimuli. This is of particular interest to applications in real time modeling and quantification of degenerative muscle pathologies and early prediction of chemical drug responses. Furthermore, the nanofiber sheets were incorporated into standard TC formats, such that the myofiber constructs could be processed and visualized using standard methods. The electrospinning set‐up and assembly process described here can be easily adapted for use in applications in which higher throughput automated imaging systems and a range of conditions are required.

Overall, the suspended elastomer nanofiber system provides a promising multifaceted microenvironment required to mimic the cellular organization of muscle, provide mechanical support, and enable assessment of skeletal muscle function, as well as a promising new tool for use in therapeutic skeletal muscle tissue engineering.

## 3 Conclusion

In this study, a biobased aliphatic polyurethane‐urea elastomer nanohybrid, P(EDS)UU‐POSS, has been synthesized. The elastomer possesses outstanding compliance, with elastic modulus of around 25 kPa and ultimate strain of 1000%. P(EDS)UU‐POSS nanofibrous sheets with uniaxially aligned orientation made by electrospinning, retain superior hyperelasticity well‐matched to the topology and physiological stiffness of native skeletal muscle. After systematic characterization of the structural, chemical, physical, and mechanical properties of the synthesized sheets, we demonstrated their suitability for use in soft tissue engineering applications. Electrospun nanofiber cell culture substrates resulted in high cell viability, and promoted cell attachment, alignment, and differentiation into skeletal muscle myotubes. Suspending the nanofibers across an acrylic microwell device enabled the hyperelastic properties of the polymer to be fully harnessed, allowing the synergistic contraction of nanofibers and human‐iPSC‐derived myofibers, and thus, the formation of an integrated hybrid muscle construct. In this form, proper sarcomeric organization was observed. Finally, SNFs were shown to support and stabilize human‐iPSC‐derived myofibers, whose functional behavior was controlled by direct optogenetic activation. Using a combination of noninvasive microscopy methods and PIV‐based image quantification, we demonstrate a method to estimate the specific force and power density exerted in the myofiber–nanofiber construct, resolving realistic values within the range of existing technologies. This novel system not only demonstrates P(EDS)UU‐POSS elastomer nanofibers as a promising new substrate for skeletal muscle tissue engineering, but also their potential to be incorporated into higher‐throughput formats for modeling and understanding the functional mechanisms of degenerative skeletal muscle pathologies.

## 4 Experimental Section

### Synthesis of Polymer

The protocol for the synthesis of the biobased polyester polyol, EDS, was kindly provided by Reverdia, The Netherlands. EDS was synthesized by polycondensation reaction between succinic acid, ethylene glycol, and diethylene glycol. Acid and hydroxyl values were measured throughout the synthesis. The final EDS product had acid value 0.39 mgKOH g^−1^ and hydroxyl value 53.2 mgKOH g^−1^. The synthesis of P(EDS)UU‐POSS polymer was adapted from the protocol of POSS‐PCLU synthesis developed previously.^[^ ^32^ ^]^ In brief, EDS polyol and trans‐cyclohexanechloroydrinisobutyl‐silsesquioxane (POSS) (Hybrid Plastics Inc., Hattiesburg, MS, USA) were placed in a reaction flask equipped with a mechanical stirrer under nitrogen. The mixture was heated to 135 °C to disperse the POSS nanocages into the polyol, until the blend became clear. 4,4′‐diisocynato dicyclohexylmethane H_12_MDI was then added to the polyol blend and reacted under N_2_ atmosphere at 70–80 °C for 2 h, forming the prepolymer. After 2 h, *N*‐dimethylacetamide (DMAC) was added dropwise to the prepolymer whilst continuously stirring. The solution was cooled to 40 °C and chain extension of the prepolymer was carried out by the dropwise addition of ethylenediamine in DMAC solution. After completion of the chain extension, 1‐butanol was used to terminate the chain extension, forming a final solution of 2% w/w POSS terminated polyurethane urea [P(EDS)UU‐POSS] in 18 w/w solid content in DMAC.

### Polymer Characterization

Number (*M*_n_) average, weight (*M*_w_) average, and peak (*M*_p_) molecular weights of the EDS polyol and P(EDS)UU‐POSS polymer were measured by GPC with a Waters Alliance GPC 2000 (Waters Corporation, Milford, MA, USA) using DMAC as eluent, and monodispersed polystyrene as the calibration standard. A flow rate of 1 mL min^−1^ was used for the eluent, with an injection volume of 5 μL. The temperature of the column was maintained at 30 °C. Samples were prepared at a concentration of 2 mg mL^−1^.

FTIR was used to identify the functional groups present in the synthesized polymer sheet. The infrared spectrum of absorption was measured between 500 and 4000 wavenumbers (cm^−1^) using a Jasco FTIR 4200. The composition of the polymer sheet was determined by identifying the typical absorbance peaks of known functional groups. Polarized FTIR was carried out by applying a polarizing filter (PL 82, Jasco, UK). Scans were generated with two different polarizer orientations, 0° and 90° which related, respectively, to parallel and perpendicular to the orientation of aligned fibers.

### Fabrication of Electrospun Nanofiber Sheets

The P(EDS)UU‐POSS polymer was precipitated in deionized water for 1 h. The DMAC residue was evaporated at 60 °C overnight. The solid polymer sheet was transferred to a glass vile and re‐dissolved in a 1:1 acetone:DMF solvent mixture yielding a final solvent concentration of 10% w/w for electrospinning. P(EDS)UU‐POSS nanofibers were produced using a custom‐built electrospinning machine developed in‐house. The electrospinning machine comprised a high voltage power supply (Glassman High Voltage) connected to a positively charged spinneret (14G tip ground‐to‐flat needle) and negatively charged collection plate, encased within an insulative chamber. The spinneret was connected, via a 25 cm length of poly(tetrafluoroethylene) (PTFE) 1/32″ tubing, to a 10 mL syringe containing the polymer solution, and positioned 20 cm above the collection plate. Constant volume flow at 1 mL h^−1^ was achieved using an automated syringe pump. The fibers were collected under a 20 kV electric field. The collection plate arrangement was modified to generate either random or aligned nanofibers. Random nanofibers were collected on a 14 × 14 cm aluminum collection plate oriented perpendicular to the needle, whilst aligned nanofibers were generated by electrostatic collection between two negatively charged metal rods, positioned 6 cm apart.

### Characterization of Morphology, Surface Properties, and Mechanical Properties of Nanofiber Sheets

The morphology of the nanofiber sheets was visualized using a Zeiss focused ion beam SEM (XB1540). The nanofiber sheets were dried for 4 h at 50 °C, mounted on 13 mm SEM stubs, and gold sputter coated using a Quorum Technologies Q150R ES Gold Coater. The samples were imaged using a 10 kV beam current. The cell seeded samples were fixed with 3% glutaraldehyde in SEM buffer (0.1 m PBS, pH 7.4 containing 0.1 m sucrose) and dehydrated in 35%, 50%, 70%, 95%, and 100% ethanol (15 min per dehydration). Samples were subsequently submerged in hexamethyldisilazane for 3 min and then air‐dried at room temperature. The samples were gold sputter coated before visualization.

The average nanofiber width and nanofiber directionality were analyzed in ImageJ. Nanofiber directionality was quantified using a FFT approach in FIJI Software. The directionality plugin was used to infer the preferred orientation of structures within the SEM image, generating a histogram of the number of structures in a given direction (−90 to +90, bins: 90). Images that had a preferred orientation, produced a histogram with a peak at that orientation. A larger peak inferred more structures with the preferred orientation.

The topology and nanophase properties of aligned electrospun nanofibers and the bulk polymer sheet was assessed by AFM in tapping mode, using an MFP‐3D system (Asylum Research, USA) with a soft 2.8 K (N M^−1^) cantilever (AC240‐R2) with a scan frequency of 1 Hz.

Tensile behavior of the bulk polymer and random and aligned nanofiber sheets were determined by tensile test applied on a universal testing machine (Instron, 5565). Dumbbell shaped samples (L: 10 mm, W: 2 mm) were elongated under 50 N load at a constant speed of 20 mm min^−1^. The average thickness of the samples was 0.044 ± 0.009 mm for the nanofiber sheets, and 0.212 ± 0.029 mm for the bulk material sheets. The force–displacement and stress–strain data were obtained from the tests using BlueHill Software. Elastic modulus, tensile strength, toughness modulus, and strain at break were calculated in Origin Software.

The effect of plasma treatment on the electrospun nanofiber sheets was determined by contact angle measurement. The water contact angle of sample with and without plasma treatment was measured using a Kruss DSA 100 Drop Shape Analyzer. For each measurement, the contact angle of a 10 μL droplet of deionized water was measured at 1, 60 s, and 1 h in room temperature conditions.

### Cell Culture and Maintenance

The C2C12 mouse myoblast cell line was cultured in Dulbecco's modified Eagle's medium (DMEM, high glucose; Thermo Fisher, 61965) supplemented with 10% fetal bovine serum (FBS; Sigma, F7524) and 1% penicillin–streptomycin (Invitrogen, 15140‐122). Once cells reached 70% confluency, the cells were passaged by trypsinization (0.25% w/v Trypsin, 2 × 10^−3^ m ethylenediaminetetraacetic acid; Invitrogen, 25200‐056) for 4 min. After cell seeding, differentiation was induced by altering the serum composition from 20% FBS, to 2% horse serum (Invitrogen, 26050070). The cultures were maintained at 37 °C, 5% CO_2_.

Human iPSCs were cultured on LN521 (Biolamina) in iPS‐Brew (StemMACS, 130‐104‐368) and passaged using TrypLE Express (Gibco, 12604013). The myofiber differentiation protocol was adapted from Rao et al.^[^ ^79^ ^]^ Briefly, iPSCs were passaged onto GFR‐Matrigel (Corning, 354230) in Essential‐6 (E6) medium (Gibco, A1516401). On day 1_pd_, the medium was replaced with E6 medium with 10 × 10^−6^ m CHIR99021 (Tocris Bioscience, 44‐231‐0). On day 3_pd_, the medium was replaced with E6 medium with 2 μg mL^−1^ doxycycline hyclate (Sigma‐Aldrich, D9891) to induce expression of the PAX7 transgene. On day 8_pd_, the medium was replaced with E6 media with 2 μg mL^−1^ doxycycline hyclate and 10 ng mL^−1^ bFGF (Sigma‐Aldrich, F3685) to enhance proliferation. This media was replaced every 2 days up to day 21_pd_. On day 21_pd_, the progenitors were split and plated on GFR‐Matrigel at 1E6 cells/in progenitor expansion media consisting of MegaCell DMEM with 5% FBS, 1% nonessential amino acid solution (SAFC, M7145), 55 × 10^−3^ m ß‐mercaptoethanol (Gibco, 21985023), 1% penicillin–streptomycin, 2 μg mL^−1^ doxycycline hyclate, and 10 ng mL^−1^ bFGF. The progenitors could be expanded and frozen at this stage. To induce md, the medium was switched to md media consisting of low‐glucose DMEM (Gibco, 11885084) with 1% N2 supplement (Gibco 17502001), 1% horse‐serum (Gibco, 26050070), and 1% penicillin–streptomycin.

### Generation of Transgenic iPAX7/ChR2‐YFP/Human iPSC Clones

Using the parental PAMV1 human iPSC line (https://www.hipsci.org/lines/#/lines/HPSI1013i‐pamv_1), a doxycycline‐inducible PAX7 transgene was stably integrated into the CLYBL safe‐harbor locus,^[^ ^80^ ^]^ by transcription activator‐like effector nucleases (TALENS) mediated gene targeting. Next, a ubiquitously expressed CAG::ChR2‐YFP vector carrying the light gated ion channel ChR2 by was inserted by PiggyBAC^[^ ^119^ ^]^ mediated transposition. A population of YFP‐expressing human iPSCs was isolated by fluorescently actuated cell sorting (FACS) using a BD‐FACS Aria III Flow Cytometer. This genetic modification enabled light‐controlled activation of contractions of human‐iPSC‐derived myofibers via blue light stimulation.

### Preparation of SNF Microwell Device for Cell Culture

For cell culture assays, the nanofibers were collected either directly onto glass coverslips or across a custom‐made acrylic microwell, termed Suspended Nanofibers (SNFs). The process of assembling SNFs is shown in Figure 4. The microwells were cut from 1.2 mm thick acrylic sheet and comprised a 12 × 12 mm^2^, with a 6.96 mm diameter well cut out of the center. One surface of the microwell was coated with double sided adhesive tape such that when the nanofibers were spun directly across the well, the nanofibers adhered to the edges of the well. The nanofibers inside the well were therefore left suspended across the well. The SNF wells were then inverted and placed inside regular 35 mm TC plastic wells. The SNFs were air plasma treated using a Zepto Diener plasma oven (0.4 mBar, 20% power, 2 min) to remove surface contaminants and improve hydrophilicity, before being UV sterilized underneath a benchtop UV lab for 15 min. Prior to cell seeding, the SNFs were incubated with 1 mL DMEM supplemented with GFR‐Matrigel (1:100) for 2 h at RT. The cells were seeded directly into the central well at a density of 30 000 cells in 100 μL media. After 5 min incubation to allow the cells to settle on the SNFs, the wells were topped up with 1.5 mL media.

### Biological Assays

Cell alignment of Phalloidin‐stained C2C12 cells was quantified on days 1, 5, and 10 for cells cultured on control wells, randomly oriented nanofibers, and aligned nanofibers. The FFT directionality plugin previously described was used. Cell viability of C2C12 cells on electrospun nanofiber sheets was tested using a live/dead assay (Invitrogen, L3224). The culture media was removed, and the cells were washed with Dulbecco's phosphate buffered saline (DPBS). A staining solution of DPBS containing 2 × 10^−6^ m calcein‐AM and 4 × 10^−6^ m ethidium homodimer (EthD‐H) was incubated with the cells for 30 min (37 °C, 5% CO_2_). The staining solution was then removed, and the cells were viewed under a Leica SP8 fluorescence microscope with 494 nm (green; calcein‐AM) and 528 nm (red; EthD‐H) excitation filters. Calcein‐AM stained the cytoplasm of live cells, and EthD‐H stained the nuclei of dead or dying cells.

Cell metabolic activity of both the C2C12 cells and human‐iPSC‐derived myofibers on the nanofiber sheets was measured using the PrestoBlue assay. The cells/nanofiber sheets were washed with PBS and incubated with a 10% solution of PrestoBlue diluted in DPBS at 37 °C, 5% CO2 for 1 h. The spent PrestoBlue solution was then transferred to a fresh black 96‐well microplate in triplicates, and absorbance at 570 nm excitation and 600 nm emission was measured on a plate reader (GloMax Discover).

### Measurement of Myofiber Contractile Activity

Contractile activity in the myofiber cultures was recorded on an Olympus IX73 microscope, fitted with a video camera. The videos were captured at 20 frames per second and were analyzed using PIVlab^[^ ^120^ ^]^ in MatLab, whereby the local displacement of small interrogation regions was analyzed between each frame of an image sequence. This enabled quantification of the contraction velocity (µm s^−1^) of spontaneous myofiber contractions. The velocity (**ν**) vectors (**ν** *_x_* and **ν** *_y_*) were exported^[^ ^84^ ^]^ from PIVLab and used to calculate displacement (**d**) vectors (**d** *_x_* and **d** *_y_*) using |**ν**| = |**d**|/*t*, and subsequent strain (*ε_x_* and *ε_y_*). Stress (**σ**) vectors were calculated using **σ** *_x_* = *ε_x_E* and **σ** *_y_* = *ε_y_E* where *E* = 30.5 kPa (average elastic modulus for cultured skeletal muscle reported in the literature). Force (**F**) vectors were estimated using **F** *_x_* = **σ** ***_x_** A_yz_* and **F** *_y_* = **σ** ***_y_** A_xz_*, where *A* was cross sectional‐area. Specific force (kN m^−2^) = stress (kPa). Power (*P*) was calculated as *P* = **F** **ν**, and power density = *P*/*V_xyz_* (where *V* = Volume) (W m^−3^).

### Immunocytochemistry

For purposes of visualization and quantification of myofiber length, width, fusion index, and directionality, the cultures were fluorescently stained. The cells were washed three times with PBS, and fixed in 4% paraformaldehyde for 30 min at RT, followed by permeabilization in PBS containing 0.1% Triton X‐100 (PBT) for 15 min at RT. The cells were then costained for F‐Actin and DNA with Phalloidin‐568 and DAPI‐405, respectively, for 60 min at RT. Reagent supplier, catalogue numbers, and working concentrations are shown in Table S1, Supporting Information. All images were taken on a fluorescence microscope (Leica SP8).

Myotube lengths and widths were measured using ImageJ software. MFI was calculated as the proportion of nuclei within myotubes with two or more nuclei, out of the total number of nuclei in the FOV. MMI was quantified as the percentage of myotubes containing five or more nuclei. For the later timepoints (day 5–10), where the cells had begun to fuse, a syncytium (or myotube) was considered as one single cell.

### Statistical Analysis

All data are presented as mean ± standard deviation (SD). Sample sizes and full details of the statistical methods carried out are detailed in Table S4, Supporting Information. Data presentation and statistical analyses were performed in Origin(Pro) Version 2021b and Graphpad Prism, Version 8.4.3 (Graphpad Software, San Diego, CA, USA, www.graphpad.com).

## Conflict of Interest

The authors declare no conflict of interest.

## Supporting information

Supporting Information

Supplemental Video 1

Supplemental Video 2

Supplemental Table 4

## Data Availability

The data that support the findings of this study are available from the corresponding author upon reasonable request.
