# Supplementary material for: Biobased Elastomer Nanofibers Guide Light‐Controlled Human‐iPSC‐Derived Skeletal Myofibers
Source: Adv Mater. 2022 Mar 31;34(18):2110441. doi: 10.1002/adma.202110441 (PMC9131876; doi:10.1002/adma.202110441)
Supplement: Supplementary file 1 — Supporting Information [file ADMA-34-0-s004.pdf]

ADVANCED MATERIALS

Supporting Information

for *Adv. Mater.,* DOI: 10.1002/adma.202110441

Biobased Elastomer Nanofibers Guide Light-Controlled Human-iPSC-Derived Skeletal Myofibers

Aimee Cheesbrough, Fabiola Sciscione, Federica Riccio, Peter Harley, Lea R'Bibo, Georgios Ziakas, Arnold Darbyshire, Ivo Lieberam, and Wenhui Song**

Supporting Information

Biobased Elastomer Nanofibers Guide Light-Controlled Human-iPSC-derived Skeletal Myofibers

Aimee Cheesbrough, Fabiola Sciscione, Federica Riccio, Peter Harley, Lea R'Bibo, Georgios Ziakas, Arnold Darbyshire, Ivo Lieberam*, Wenhui Song**

List of abbreviations:

3D	Three-dimensional
AFM	Atomic force microscopy
ALS	Amyotrophic Lateral Sclerosis
ChR2	Channelrhodopsin-2
CNS	Central nervous system
DMD	Duchenne Muscular Dystrophy
DR	Dichroic ratio
ECM	Extracellular matrix
ESC	Embryonic stem cell
FTIR	Fourier transform infrared spectroscopy
FTT	Fast Fourier transform
GPC	Gel permeation chromatography
ICC	Immunocytochemistry
IF	Immunofluorescence
iPSC	induced pluripotent stem cells
MFI	Myotube fusion index
MMI	Myotube maturation index
MN	Motor neuron
PIV	Particle image Velocimetry
PU	Polyurethane
PUU	Polyurethane urea
SD	Standard deviation
SEM	Scanning electron microscopy
SM	Skeletal muscle
SNFs	Suspended Nanofibers
TC	Tissue culture
TPU	Thermoplastic Polyurethane

List of symbols:

σ	Stress
ν	Velocity
V	Volume
ε	Strain
E	Elastic Modulus
M_n	Number average molecular weight
M_p	Molecular weight of highest peak
M_w	Weight average molecular weight
d	Displacement
P	Power

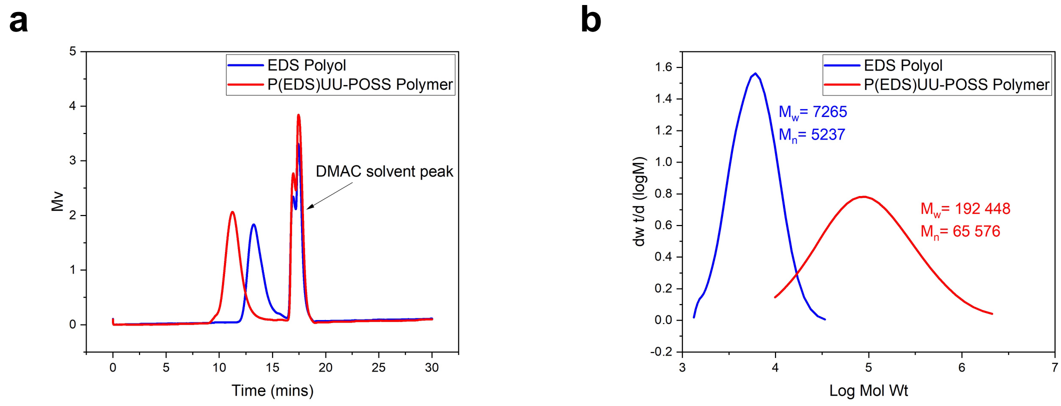

Figure S1 Gel Permeation Chromatography (GPC) (a) Overlayed chromatogram plot and (b) molecular weight distribution of EDS polyol and P(EDS)UU-POSS polymer samples.

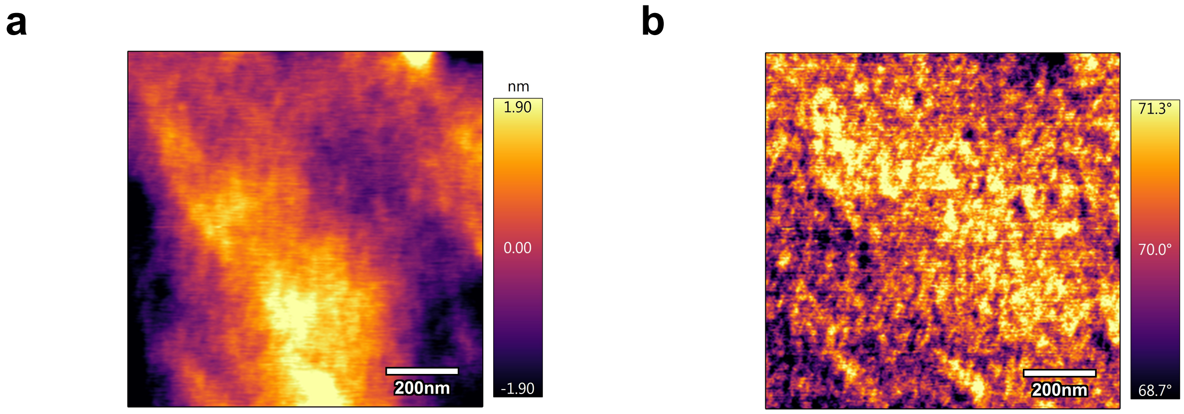

Figure S2 Atomic force microscopy (AFM) (a) Height and (b) phase images of cast polymer sheet.

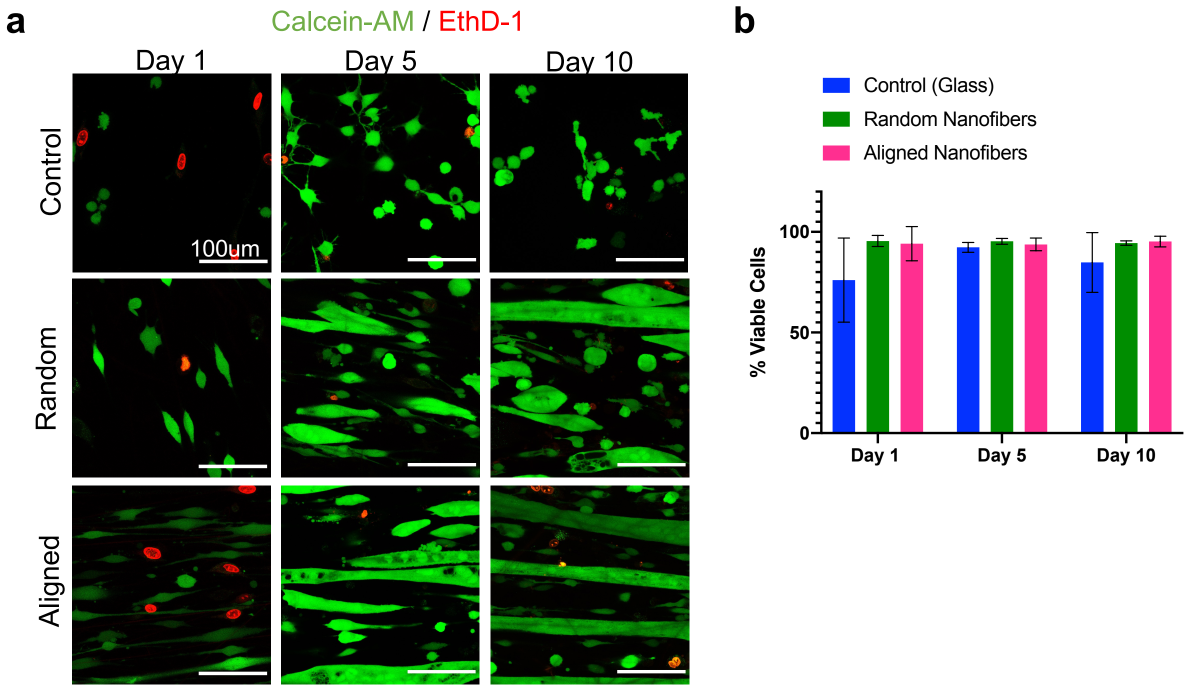

Figure S3. Live/Dead Assay on C2C12 cultures. (a) Immunocytochemistry (ICC) images representative of the viability (Calcein-AM) and cytoxicity (Ethidium Homodimer, EthD-1) of C2C12 cells cultured on glass coverslips, and on coverslips coated with random and aligned nanofibers on day 1, 5 and 10 days in culture. Scale bars: 100um. (b) Live/Dead Assay Quantification**.** Percentage of viable cells quantified from Live/Dead stained ICC images. For the later timepoints (day 5-10), where the cells had begun to fuse, a syncytium (or myotube) was considered 1 single cell.

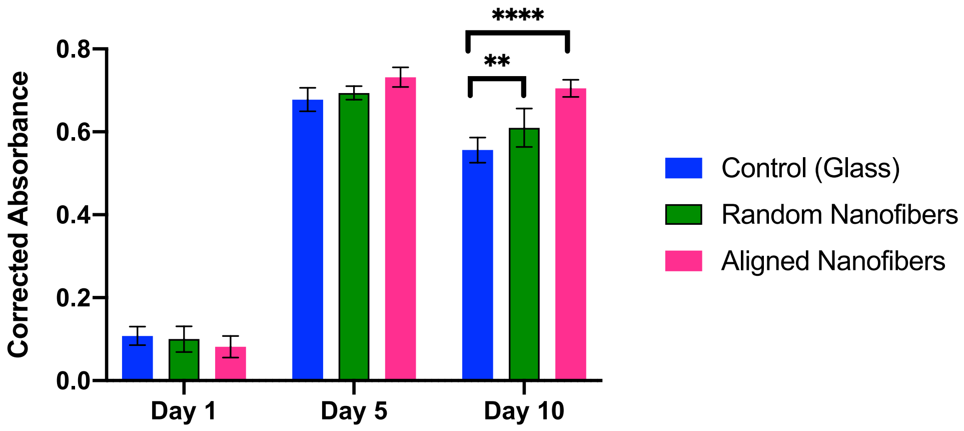

Statistical significance tests shown in Table S3.

Figure S4. PrestoBlue Assay for Metabolic Activity. Absorbance readings of 10% PrestoBlue solution after 1hr incubation with C2C12 cells cultured on glass coverslips, and on coverslips coated with random and aligned nanofibers on day 1, 5 and 10 days in culture. Statistical tests shown in Table S4.

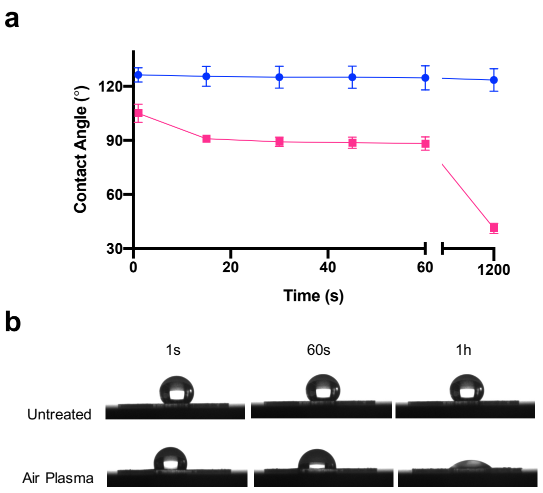

Figure S5. Air plasma treatment improves surface hydrophilicity of nanofiber sheets. (a) Surface contact angle measurements and (b) water droplet images at 1s, 60s and 1h for Untreated and Air Plasma-treated POSS-EDS-PU Electrospun nanofiber sheets.

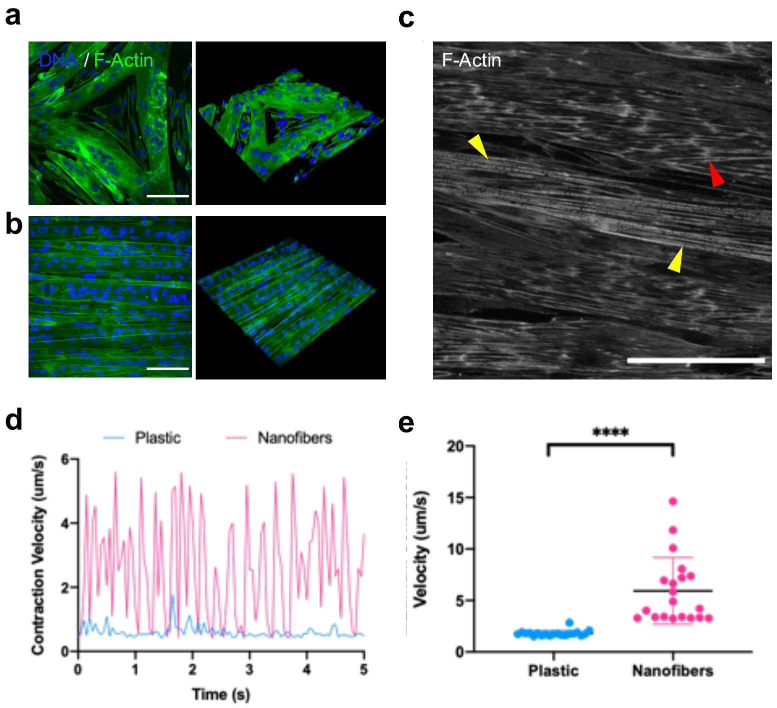

Figure S6. Enhanced Sarcomere organization leads to spontaneous contractile activity in C2C12 myofibers on Suspended Nanofiber Sheets (SNFs). Immunocytochemistry (ICC) Images and 3D rendering of C2C12 myofibers cultured on (a) Tissue culture (TC) plastic and (b) Suspended Nanofibers (SNFs). (c) Organized filamentous actin (F-Actin) observed only in myofibers on SNFs. Assembly of myofibrils visualized as continuous striated actin filaments (Yellow arrowheads). Sarcomere-like periodic patterning (Red arrowhead) observed as F-Actin clustering at the Z-line. (d) Particle Image Velocimetry (PIV) trace of spontaneous contraction (no electrical or optogenetic stimulation) velocity and (e) Maximum contraction velocity, observed in Day 14 C2C12 Myofibers on TC plastic and on SNFs. SB: 100μm.

a

b

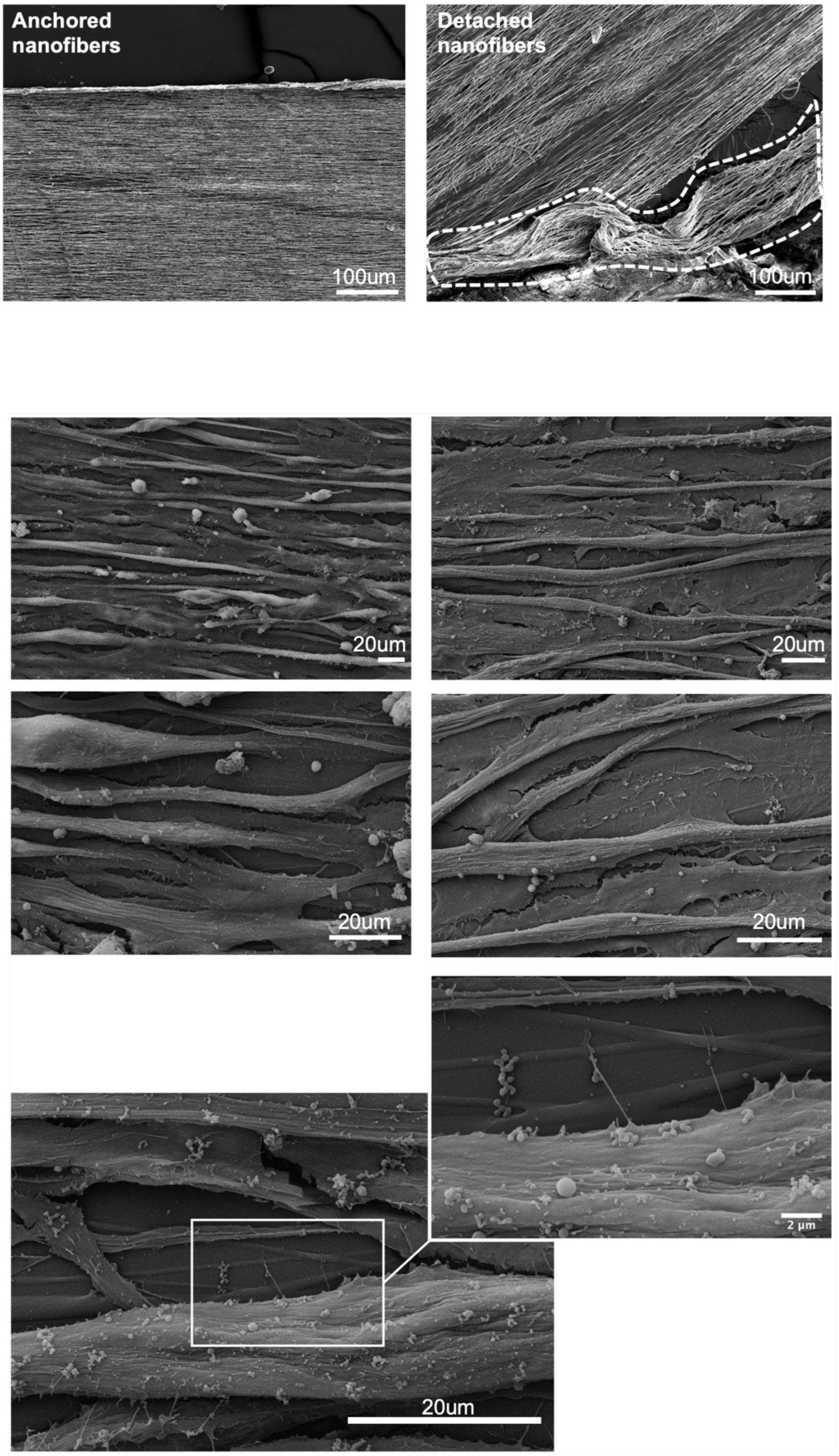

Figure S7. Scanning Electron Microscopy (SEM) images of (a) Aligned electrospun nanofiber sheets. Thin nanofiber sheets collected directly onto formats for cell culture are anchored and maintain their aligned arrangement. Nanofibers which are removed from their collection substrate can curl/fold up and lose their uniform arrangement. (b) Human iPSC-derived myofibers on Suspended Nanofibers (SNFs).

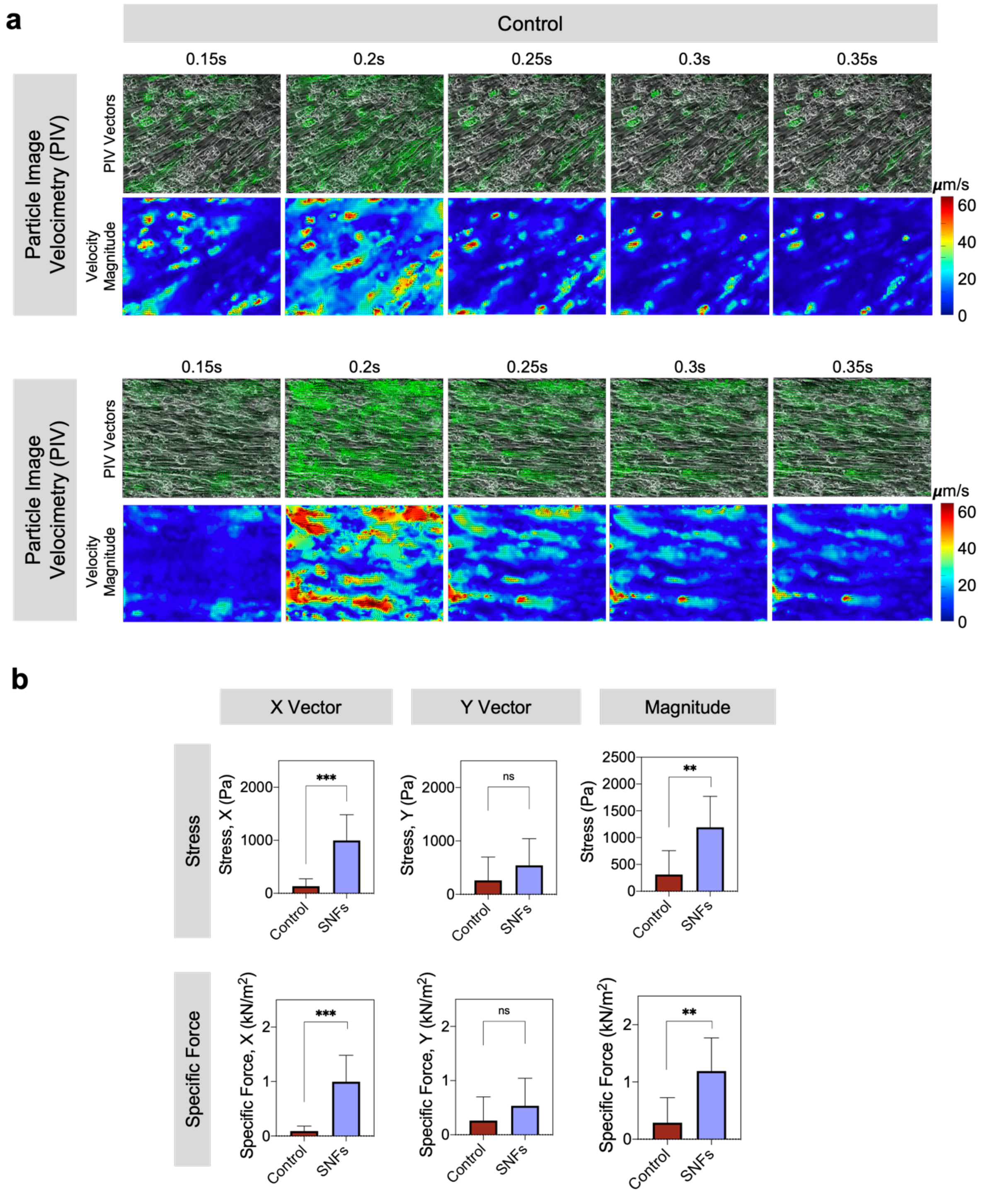

Figure S8. (a) Particle image Velocimetry (PIVLab) plots of synergistic human iPSC-derived myofiber-nanofiber contractions, controlled by optogenetic activation. Peak contraction occurs at 0.02s in both control and nanofibers samples. (b) Calculated X, Y and magnitude of stress and specific force at day 20.

Table S1. List of products, manufacturers/suppliers and catalogue numbers used for polymer synthesis, cell culture and experimental procedures.

	Product	Manufacturer/Supplier	Catalogue
Chemicals and solvents	Succinic acid, BioXtra, >99.0%	Sigma-Aldrich	S3674
	Ethylene glycol, 99.8%	Sigma-Aldrich	324558
	Diethylene glycol, ReagentPlus, 99.0%	Sigma-Aldrich	H26456
	Transcyclohexanechloroydrinisobutyl-Silsesquioxane (POSS)	Hybrid Plastic Inc. Hattiesburgs, United States	AL0125CH
	4,4' diisocynato dicyclohexylmethane ($H_{12}MDI$)	Evonik Operation GmnH	3007714724
	n-dimethylacetamide (DMAC), 99.8%	Sigma-Aldrich	271012
	Ethylenediamine, ReagentPlus, >99%	Sigma-Aldrich	E26266
	1-butanol, 99.8%	Sigma-Aldrich	281549
	Acetone, 99.6%	Acros Organics	423240010
	N,N-Dimethylformamide (DMF), 99.8%	Acros Organics	326870010
	Glutaraldehyde solution, Grade I, 25% in H_2O	Sigma-Aldrich	G5882
	Ethanol, BioUltra, >99.8%	Sigma-Aldrich	51976
Cell Culture	Dulbecco's Modified Eagle Medium (DMEM), high glucose	Thermofisher	61965
	Fetal bovine serum (FBS)	Sigma-Aldrich	F7524
	Penicillin-streptomycin	Invitrogen	15140-122
	Trypsin-EDTA	Invitrogen	25200-056
	Horse serum	Invitrogen	26050070
	Laminin521 (LN521)	Biolamina	LN521
	TrypLE™ Express	Gibco	12604013
	Growth Factor Reduced (GFR)-Matrigel™	Corning	354230
	Essential-6™ (E6) Medium	Gibco	A1516401
	CHIR99021	Tocris Bioscience™	44-231-0
	Doxycycline hyclate	Sigma-Aldrich	D9891
	bFGF	Sigma-Aldrich	F3685
	MegaCell DMEM™	SAFC	M3942
	Non-essential amino acid solution	SAFC	M7145
	ß-mercaptoethanol	Gibco	21986023
	Low glucose DMEM	Gibco	11885084
	N2 supplement	Gibco	17502001
ICC[i]/Cell viability	F-Actin/Alexa Fluor™ 568 Phalloidin (1:1000)	Invitrogen	A12380
	DNA/DAPI (300nM)	Invitrogen	D1306
	TTN (Titin)/9D10 (2ug/ml)	DSHB	9D10
	LIVE/DEAD™ Viability/Cytotoxicity (2uM Calcein-AM, 4uM EthD-H)	Invitrogen	L3224
	PrestoBlue™ Cell Viability Reagent (1:10)	Invitrogen	A13261

i) Immunocytochemistry

Table S2. Analysis of Polarized FTIR Spectra of Random and Aligned Nanofibers.

Wavenumber (cm^{-1})	Functional Group	Random Nanofibers			Aligned Nanofibers		
		Dichroic Ratio, DR	Estimated α Interval	f_m	Dichroic Ratio, DR	Estimated α Interval	f_m
1042	COC, OCC, Si-O-Si	0.988	$54.90° \leq \alpha \leq 90°$	0.008	1.911	$0° \leq \alpha \leq 45.65°$	0.232
1140	COC, OCC, Si-O-Si	0.979	$55.03° \leq \alpha \leq 90°$	0.014	1.772	$0° \leq \alpha \leq 46.73°$	0.205
1213	COC, OCC, Si-O-Si	0.982	$54.98° \leq \alpha \leq 90°$	0.012	1.583	$0° \leq \alpha \leq 48.34°$	0.163
1715	C=O, COO, NCO, $CO(NH)_2$	0.977	$55.05° \leq \alpha \leq 90°$	0.015	1.484	$0° \leq \alpha \leq 49.26°$	0.139
2838	CH2	1.020	$0° \leq \alpha \leq 55.47$	0.006	1.391	$0° \leq \alpha \leq 50.18°$	0.115
2919	CH2	0.987	$54.91° \leq \alpha \leq 90°$	0.008	1.311	$0° \leq \alpha \leq 51.0°$	0.094

α = Estimation interval value, f_m= minimum fractions of molecular segments

$$\text{If } DR > 1: \quad 0° \leq \alpha \leq arccot\left(\frac{1}{2}\sqrt{DR}\right); \qquad f_m = \frac{DR-1}{DR+2}$$

Equation (S1)

$$\text{If } DR < 1: \quad arccot\left(\frac{1}{2}\sqrt{DR}\right) \leq \alpha \leq 90°; \qquad f_m = \frac{2(1-DR)}{DR+2}$$

Equation (S2)

Table S3. Table of existing literature reporting methods to stabilise and measure contractile forces exerted in tissue engineered skeletal muscle.

Authors	Cell type	ECM/Support Description	Culture duration	Specific Force (kN/m^2)	Stimulation Method	Measurement tool
Dennis and Kosnik, (2000) [101]	Primary rat myoblasts, satellite cells and fibroblasts	Myooid: PDMS coated plate with anchor points	45-50 days	2.9	Electrical	Force transducer (direct)
Dennis et al. (2001) [102]	Mouse skeletal myoblasts (C2C12) + 10T1/2 Fibroblasts	Myooid: PDMS coated plate + silk suture anchors	30 days	4.5	Electrical	Force transducer (direct)
Huang et al. (2005) [103]	Primary rat myoblasts	Myooid: PDMS + Silk suture anchors + fibrin gel	14 days	36.3	Electrical	Force transducer (direct)
Yan et al. (2007) [104]	Primary rat myoblasts	Collagen I painted fibrils	21 days	0.95+/-0.4	Electrical	Force transducer (direct)
Liao et al. (2008) [105]	Mouse skeletal myoblasts (C2C12)	PU nanofibers	14 days	2.2 †	Electrical	Force transducer (direct)
Hinds et al. (2011) [86]	Primary rat myoblasts	Collagen I, fibrin, Matrigel hydrogel, in silicone mould + Velcro anchor points.	14 days	5.5 ± 0.6 - 9.4 ± 0.7	Electrical	Force transducer (direct)
Khodabukus and Baar. (2012) [106]	Mouse skeletal myoblasts (C2C12) and 3T3 Fibroblast	Myooid: PDMS + Silk suture anchors + fibrin gel	14 days	0.4-0.8	Electrical	Force transducer (direct)
Stasko et al. (2013) [107]	Skeletal muscle (diaphragm)	Ex-vivo whole skeletal muscle bundles	N/A	0.03-0.21	Electrical	Force transducer (direct)
Carosio et al. (2013) [108]	Primary mouse myoblasts	Delaminated monolayer, steel pin anchored	15-40 days	0.893+/- 0.110	Electrical	Force transducer (direct)
Sato et al. (2013) [109]	Mouse skeletal myoblasts (C2C12) +-Gene transfer,	Magne-TE muscle-ring, steel pin anchored	7 days	0.97-1.02	Electrical	Force transducer (direct)
Morimoto et al. (2013) [110]	C2C12 + primary mouse neural stem cells (mNSCs) co-culture	Striped Matrigel patterning + PDMS stamp, fibronectin anchor points	18 days	0.2-3.5 *	Electrical	Micro-post displacement (indirect)
Ito et al. (2014) [111]	Mouse skeletal myoblasts (C2C12)	Magne-TE muscle ring + Collagen I/Matrigel hydrogel, steel pin anchored	10 days	0.01 (gel only), 0.03 (Magne-TE + gel) *	Electrical	Force transducer (direct)
Juhas et al. (2014) [95]	Rat primary myoblasts	Collagen I, fibrin, Matrigel hydrogel, in silicone mould + Velcro anchor points.	14 days	47.9	Electrical	Force transducer (direct)
Khodabukus et al. (2015) [112]	Mouse skeletal myoblasts (C2C12)	Myooid: PDMS + Silk suture anchors + fibrin gel	14 days	0.1-0.3	Electrical	Force transducer (direct)
Uzel et al. (2016) [113, 114]	Mouse skeletal myoblasts (C2C12) + ChR2+ iPSC-derived MNs	PDMS microfluid co-culture device, micro-posts, Collagen I/Matrigel hydrogel	16 days	0.04kN/m^2	Electrical and Optogenetic	Micro-post displacement (indirect)
Rao et al. (2018) [79]	Human iPSCs, induced PAX7	3D Myobundles anchored within nylon frame	28 days	0.8-3	Electrical and Chemical (Acetylcholine)	Force transducer (direct)
Osaki et al. (2018) [18]	Human iPSC-derived skeletal myoblasts (+/- MN Coculture)	Collagen/Matrigel-myoblast mix, pillar anchored, microfluid device	14 days	0.25 (Muscle only)	Electrical (Muscle only) and Optogenetic (+MNs)	Micro-post displacement (indirect)
Khodabukus et al. (2019) [115]	Human primary myoblasts	3D Myobundles anchored within nylon frame	7 days	9.1 ± 0.38 - 19.3 ± 0.63	Electrical	Force transducer (direct)
Vila et al. (2019) [114, 116]	Human primary myoblasts + ChR2+ iPSC-derived MNs	PDMS microfluid co-culture device, micro-posts, Collagen I/Matrigel hydrogel	20 days	0.193 kN/m^2 * †	Electrical and optogenetic	Micro-post displacement (indirect)
Nagashima et al. (2020) [117]	Human myoblasts (Hu5/KD3)	Dumbell-shaped microwell + PDMS flexible posts	8 days	0.226	Electrical	Micro-post displacement (indirect)
Afshar et al. (2020) [100]	Human primary myoblasts	MyoTACTIC: 96-well PDMS micro-posts	7-14 days	1-5 *	Electrical and Chemical (Acetylcholine)	Micro-post displacement (indirect)
Yoshioka et al. (2021) [118]	C2C12, or human iPSCs induced MyoD	Magne-TE system, PDMS micro-posts	10 days	0.0089- 0.016	Electrical	Micro-post displacement (indirect)
**This study: Cheesbrough et al. (2022)**	**Human iPSC, induced PAX7**	**Suspended elastomer nanofibers**	**20 days**	**1.19+/-0.58**	**Optogenetic**	**Muscle sheet displacement, PIV (indirect)**

(*) = Value estimated from figure, (†) = Estimate calculated from reported force and device/construct dimensions.

Table S4. Table of statistical tests performed, significance levels and p-values available from the Wiley Online Library.

Supporting videos: Video 1 and Video 2 are available from the Wiley Online Library.
